# Organizational Structure, Performance Monitoring, and Academic Staff Performance in Selected Private Chartered Universities: A Qualitative Study

**DOI:** 10.12688/openresafrica.15891.3

**Published:** 2025-12-18

**Authors:** Turyamureeba Silaji, Zulaihatu Lawal Bagiwa, Tukur Muhammad

**Affiliations:** 1 Department of Educational Foundation, Faculty of Education,, Kampala International University - Western Campus, Bushenyi, Western Region, Uganda; 2 Department of Science Education, Faculty of Education, Kampala International University - Western Campus, Bushenyi, Western Region, Uganda

**Keywords:** Organisational Structure, Performance Monitoring, Academic Staff Performance, Private Chartered Universities:

## Abstract

This qualitative study investigates how organizational structure and performance monitoring influence academic staff performance in selected private chartered universities in Western Uganda. Using a phenomenological design, the study explored the lived experiences and perceptions of 10 academic leaders, including deans and senior lecturers, across various faculties. Participants were purposively selected for their strategic roles in academic administration, with qualifications ranging from Master’s degrees to PhDs in fields such as Education Management, Science Education, and Clinical Medicine. Data were collected through in-depth interviews and thematically analyzed. Findings revealed that highly centralized structures, lack of autonomy, and inconsistent monitoring mechanisms contribute to diminished staff morale and reduced teaching effectiveness. However, faculties led by deans with advanced academic qualifications and more inclusive leadership approaches demonstrated stronger performance cultures, improved staff engagement, and better alignment with institutional goals. The study highlights the critical role of academic leadership, structural clarity, and transparent performance monitoring in enhancing academic productivity. It recommends rethinking internal governance frameworks to foster participatory management and accountability within Uganda's private university sector.

## 1.0 Background to the study

The performance of academic staff is central to the quality and effectiveness of higher education institutions. It directly influences teaching outcomes, research productivity, and student learning experiences ( Altbach, 2015). In Uganda, private chartered universities have grown significantly over the past two decades, providing increased access to higher education. As highlighted by Mushemeza (2016), academic staff in African universities often face structural and motivational challenges that affect their effectiveness. Whereas Tibarimbasa (2010) found that ineffective management structures significantly affect performance in private universities in Uganda. However, this expansion has brought with it governance and administrative challenges that may compromise academic staff performance, especially in areas related to institutional structure and internal monitoring systems ( Nakimuli & Aguti, 2020).

Organisational structure refers to the formal arrangement of roles, responsibilities, communication lines, and authority within an institution. A functional structure fosters accountability, clarity in task execution, and efficient decision-making ( Mintzberg, 1983). In many private universities, however, decision-making processes remain highly centralized, with limited delegation of authority and low involvement of academic staff in governance processes ( Ssekamwa, 2018). This centralization often results in low staff motivation, role conflict, and diminished autonomy, which ultimately impacts academic output.

In parallel, performance monitoring—defined as the systematic tracking and evaluation of staff activities against institutional goals—is another critical determinant of staff effectiveness. Effective performance monitoring promotes transparency, goal alignment, and continuous improvement ( Armstrong, 2020). Yet, in several private institutions, monitoring systems are inconsistently applied, and performance evaluations are often irregular, subjective, or linked to managerial discretion rather than evidence-based metrics ( Kasozi, 2009).

Despite the growing body of literature on academic staff performance in public universities, there is limited research focusing on how organisational structures and internal monitoring mechanisms affect performance in Uganda’s private university sector. This study seeks to address this gap by exploring the lived experiences and perceptions of academic leaders in selected private chartered universities in Western Uganda. Understanding these dynamics is essential for developing governance reforms and institutional policies that can enhance performance and ensure sustainable academic quality.

## 1.2 Theoretical grounding

While the foundational principles of organizational theory (e.g., Mintzberg) and performance management (e.g., Armstrong) define the central variables of this study, a deeper analysis of the observed relationships necessitates the integration of three complementary models. First, Contingency Theory is employed to analyze the organizational structure, positing that its effectiveness depends on the fit between the structure and the private university’s specific operational and regulatory environment. This lens helps interpret why certain structural rigidity persists and how it facilitates or inhibits academic objectives. Second, Herzberg’s Motivation-Hygiene Theory is critical for interpreting the outcomes of the performance monitoring system. This theory distinguishes between factors that prevent dissatisfaction (Hygiene Factors, e.g., salary, working conditions) and those that actively drive excellence (Motivator Factors, e.g., recognition, achievement). We use this to explain why staff may be compliant but not necessarily motivated for high performance. Finally, Organizational Justice Theory provides the framework for examining staff perceptions of the monitoring system by focusing on the fairness of the procedures (procedural justice), the outcomes (distributive justice), and the interpersonal treatment (interactional justice). Utilizing these three theories elevates the analytical rigor, allowing the discussion to move beyond description to interpret the impact and meaning of the findings within established theoretical discourse.

## 1.1 Methodology

### Research design

This study employed a qualitative phenomenological design, aimed at exploring and interpreting the lived experiences of academic staff and leaders concerning how organisational structure and performance monitoring influence academic performance. The phenomenological approach was chosen to allow for in-depth understanding of individual perspectives within their real-world institutional contexts ( Creswell, 2013).

### 1.1.1 Study area and population

The study was conducted in two private chartered universities located in Western Uganda. These universities were selected because they represent a cross-section of the region's private higher education landscape, each with established academic structures and internal monitoring systems. The study population consisted of academic leaders, including deans, heads of departments, and senior lecturers with administrative responsibilities.

### 1.1.2 Sampling procedure and sample size

A purposive sampling technique was employed to select individuals with substantial insight into institutional governance and academic performance processes. A total of 10 participants were selected, comprising academic leaders with postgraduate qualifications in fields such as Education Management, Curriculum Studies, Clinical Medicine, and Science Education. This sample ensured depth and diversity of views relevant to the study objectives.

### 1.1.3 Data collection methods

Semi-structured interviews were used to collect qualitative data. An interview guide was developed to cover key themes such as organisational structure, decision-making, communication flow, performance appraisal, and staff motivation. Interviews were conducted face-to-face, lasting approximately 20–30 minutes each. All interviews were audio-recorded with the participants’ consent and supplemented with field notes to capture non-verbal cues and contextual information.

### 1.1.4 Data analysis

Interview recordings were transcribed verbatim and analyzed thematically following the six-step approach outlined by Braun & Clarke (2006). The process involved coding textual data, identifying recurring patterns, and developing themes that captured the essence of participants' experiences. NVivo software was used to support the organization and retrieval of data. To ensure trustworthiness, techniques such as member checking, peer debriefing, and maintaining an audit trail were applied.

### 1.1.5 Ethical approval statement

This study received ethical approval from the **Research Ethics Committee of Kampala International University**, Uganda. The approval was granted on **September 6 ^th^ 2024**, with the reference number KIU-2024-292. The ethics committee approved the research protocol, participant recruitment procedures, and data protection measures. Also, Research Ethics Committee approved the use of **verbal informed consent** due to the minimal risk nature of the study and literacy considerations among some participants. The manuscript has been updated to include the ethical approval date. The Uganda National Council for Science and Technology (UNCST) granted ethical approval on **8 ^th^ October 2024**, under national approval number **SS3145ES**. uncst.go.ug

### 4.5.3 Thematic findings aligned with research objectives

**Figure 1.**
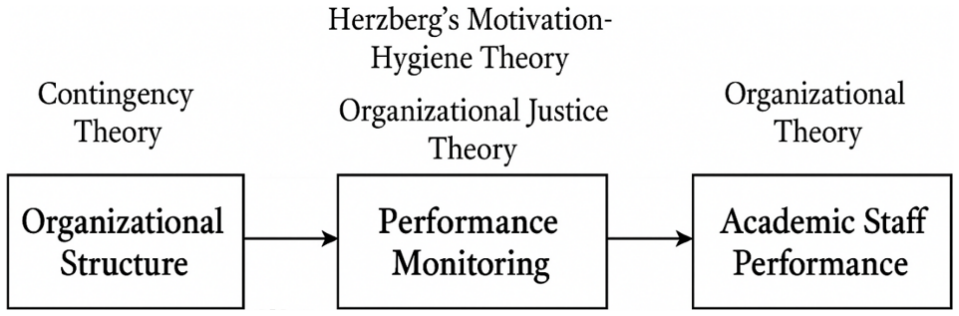
Conceptual Framework: The Mediation of Organizational Justice and Motivation on Academic Staff Performance. **Figure 1** illustrates the logical flow and theoretical foundation underpinning how **organizational structure** and **performance monitoring** influence **academic staff performance**, with **organizational justice** and **motivation** serving as mediating mechanisms.

Responses from selected participants (interviewees) on Organizational Structure (Theme 1), Performance Monitoring (Theme 2), Relationship and Perception of Academic Staff on Performance Monitoring (Theme 3), Academic Staff Performance (theme 4), and General Feedback and Recommendations (Theme 5) in chartered universities in western Uganda qualitatively reported below ( Figure 1).

***4.2.4.1 Theme 1; Organizational structure***

**Figure 2.**
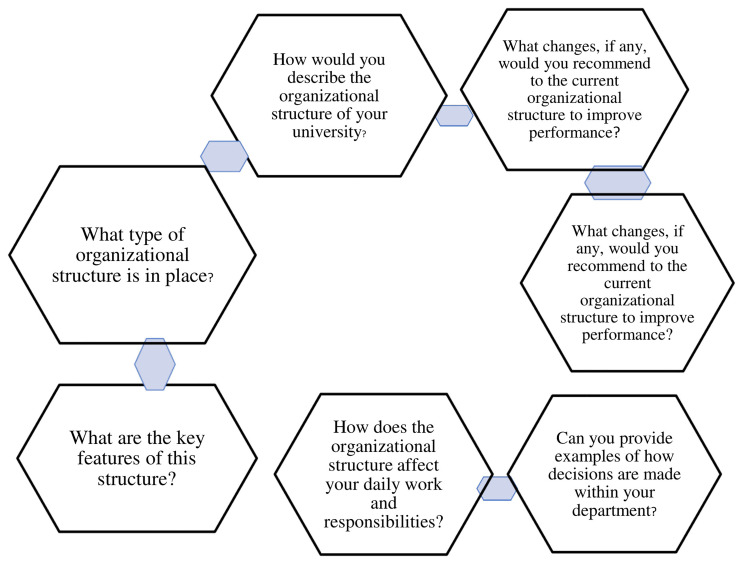
shows theme 1, Organizational Structure.

Explores the opinions of deans of faculties of private universities in western Uganda on the organizational structure. Under this theme are seven (7) sub-themes as depicted in Figure 2.

How would you describe the organizational structure of your university?

Participants described the organizational structure as hierarchical and bureaucratic, characterized by formalized procedures and a clearly defined chain of command. The structure is composed of various levels:

Top Levels: Board of Trustees → Council → Top Management.

Top Management Members: Vice Chancellor, Academic Registrar, Dean of Students, Director of Quality Assurance, Director of Graduate Studies, Librarian.

Lower Levels: Faculty Deans → Heads of Departments.

Most universities employ a hybrid model that combines both centralized and decentralized elements. The centralized aspects are reflected in hierarchical reporting structures, such as the Senate and Vice-Chancellor, while the decentralized features empower departments to make decisions within their functional areas.

DU PT2-86… "It’s an eclectic model—some decisions are made at lower levels; others require top approval."

DU PT4-163…"Departments operate autonomously but align with institutional policies."

DU PT2-87 echoed on Centralized features include hierarchical reporting (e.g., Senate, Vice Chancellor), while decentralized aspects empower departments

“It is a bureaucratic system. Top levels include the Board of Trustees, Council, and Top Management, while the lower levels consist of Faculty Deans and Heads of Departments.”

 DU PT2-86

What are the key features of this structure?

Participants from private universities in Western Uganda described the organizational structure as comprising bureaucratic systems, decentralized structures, and hybrid models. The bureaucratic structure is characterized by formalized processes that require decisions to pass through established channels before approval. While this structure ensures order, consistency, and accountability, it can also slow down decision-making processes, particularly when higher-level authorization is required. The decentralized structure allows departments to operate autonomously, making decisions within their functional areas without constantly seeking approval from higher authorities. This approach promotes efficiency and timely decision-making at the departmental level.

Additionally, some universities adopt a hybrid model, which combines decentralized daily operations with centralized policy-making. Under this model, departments enjoy operational autonomy, while overarching policies and strategic decisions are made at higher levels of governance, such as the University Council or Vice Chancellor’s office.

DU PT4-162 "Each department is autonomous and can make decisions independently"

DU PT2-88 "Decisions have to be approved by the Senate... it is bureaucratic"

“Bureaucratic structure causes delays, especially when required personnel are unavailable for approvals. Processes are slowed down by the need to follow formal channels.”

DU PT9-362

How does the organizational structure affect your daily work and responsibilities?

Participants reported that the bureaucratic system has both positive and negative effects. On the positive side, the bureaucratic structure provides clear accountability and well-defined role delegation, ensuring that responsibilities are properly assigned and monitored. However, this structure also affects daily operations by causing delays due to the need for formal approvals and strict adherence to established protocols. Academic staff members often have to wait for decisions from higher authorities, which can hinder efficiency and slow down important processes.

“The bureaucratic structure causes delays, especially when required personnel are unavailable for approvals.” DU PT2-82

"The structure Makes me more committed" DU PT6-283

Can you provide examples of how decisions are made within your department?

The interviewees asserted that decision-making is typically structured and follows a top-down approach. However, departmental decisions often begin at lower levels before being escalated to top management for approval.

“Departmental teams meet, reach consensus, and forward requests to the Dean. The dean endorses requests, which are then sent to top management for approval.”

DU PT4-174 Similar findings were reported by Nganga and Mwaura (2021) in Kenya, where centralization in private universities impeded academic performance. According to Silaji *et al.* (2025b), hybrid governance models offer a balance between institutional control and departmental autonomy.

How does the organizational structure support or hinder collaboration among academic staff?

The participant reported that the organizational structure can both support and hinder collaboration among academic staff. Decentralized elements and hybrid models promote collaboration by allowing quick decision-making and operational autonomy at the departmental level. However, centralized bureaucratic systems can hinder collaboration by causing delays due to formalized procedures and slow approval processes. Poor communication between top management and departments further reduces effective collaboration. A balanced structure that combines guidance from top management with departmental autonomy can enhance collaboration.

What changes, if any, would you recommend to the current organizational structure to improve performance?

The participants suggested that decentralization could enhance efficiency by reducing delays associated with the bureaucratic process.

“I suggest that decentralization could improve efficiency.”

DU PT4-165

### Analysis summary

The findings indicate that while the bureaucratic structure provides a clear framework for decision-making and accountability, it also leads to delays and inefficiencies. The recommendation for decentralization aligns with the need for quicker decision-making and more effective performance monitoring.

***4.2.4.2 Theme 2 performance monitoring***

**Figure 3.**
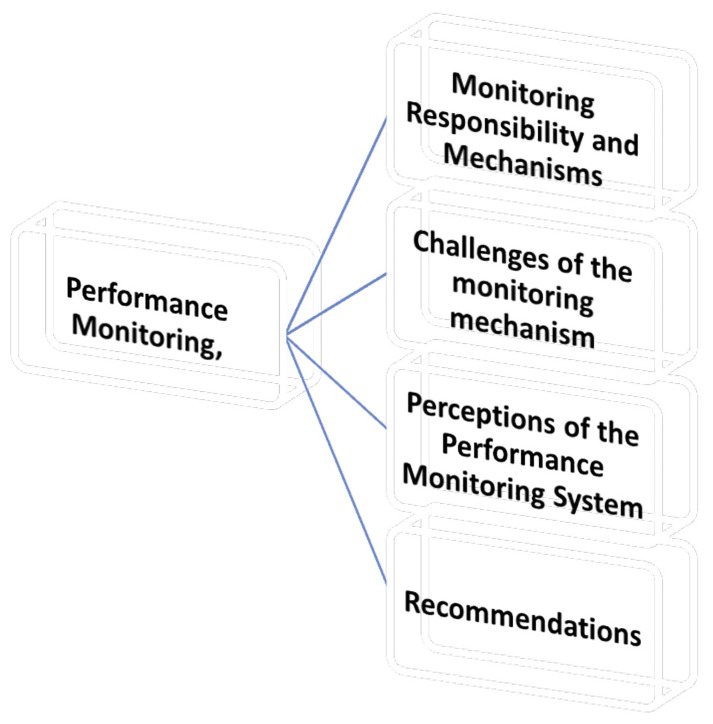
Shows the Performance Monitoring.

This section explores how academic performance monitoring is conducted within the university, focusing on the procedures, criteria, frequency, challenges, and suggested improvements reported by the Deans. Under this theme are four (4) sub-themes as shown in Figure 3.

Participants submitted that the director of quality assurance is responsible for monitoring academic staff performance. Participants have indicated a lack of biometric machines for monitoring, resulting in non-automated oversight. Performance evaluations occur during scheduled appraisals, with Faculty Deans receiving monthly reports from Heads of Departments outlining both achievements and outstanding tasks. The primary tools for annual assessments include biometric attendance records and student feedback.

*"Quality assurance checklists focus on attendance, not motivation."*

DU PT6-226

Challenges of the monitoring mechanism

Participants asserted that there is inconsistent implementation, bias in student feedback

……………… *Biometric systems abandoned post-COVID*

DU PT2-99 …… *"Only 30% of students submit evaluations*”

Oonyu and Opolot-Okurut (2019) observed that effective performance monitoring enhances staff motivation and institutional commitment. Perceptions of the Performance Monitoring System

The participants consider the monitoring system generally fair but note that overwhelming responsibilities can hinder its effectiveness. Performance monitoring directly influences motivation through payment processing. PT5 described the system as *"fair and transparent; minimal complaints,"* suggesting that when the system works as intended, it is generally perceived as equitable.

*“Timely payment on the 25th of each month serves as a motivator”*

DU PT6-269

A significant concern was the bias and low response rates related to student feedback, which was perceived as incomplete and often skewed. 

*"Student feedback often incomplete or skewed,"*

DU PT6-227

As highlighted by DU PT6-320, reflecting a sense that the feedback gathered from students does not provide a full picture of the system's impact. Additionally, overemphasis on attendance was identified as a limitation of the monitoring system.

"The system focuses on attendance, not inner drive,"

DU PT6-228

DU PT6-316 remarked, criticizing the system for prioritizing physical presence over more intrinsic factors such as motivation and engagement.

This indicates that while the system is seen as fair, it may lack the motivational elements necessary to drive sustained engagement and improvement in student performance.

Recommendations

Participants indicated that the feedback on the monitoring system highlighted both challenges and suggestions for improvement. DU PT5-192 suggested automating monitoring, such as reintroducing biometric systems, to improve accuracy and efficiency. Additionally, DU PT2-111 recommended establishing focus groups to ensure balanced and comprehensive feedback. The evaluation process involves students providing feedback on lecturers' teaching effectiveness, assessment practices, and attendance, which is then communicated to academic staff for improvements. However, several challenges were identified, including a lack of daily monitoring due to heavy workloads, the absence of a dedicated department for performance monitoring, and inconsistencies in existing monitoring systems.

To address these issues, it was suggested that a dedicated monitoring office be established to enhance the efficiency, consistency, and fairness of the performance monitoring process.

***4.2.4.3 Theme 3: Relationship and perception of academic staff on performance monitoring***

**Figure 4.**
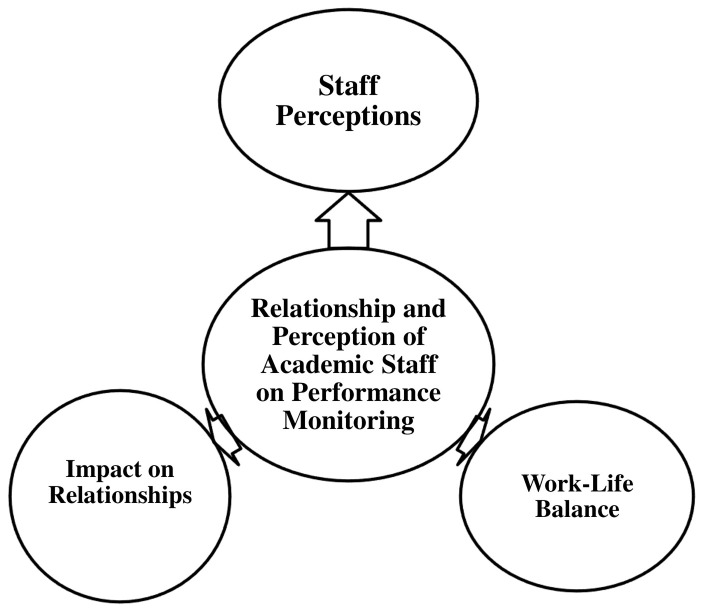
Relationship and Perception of Academic Staff on Performance Monitoring.

This section examines the relationship between academic staff and performance monitoring within the university, with a particular focus on staff perceptions. It explores how performance monitoring influences workplace relationships and work-life balance. This theme is further divided into three sub-themes, as illustrated in Figure 4.

**Staff Perceptions**

Participants had mixed views on the monitoring system. On the positive side, DU PT2-107 shared that the system "motivates me to improve," suggesting that, for some, the monitoring process can act as a motivating factor, encouraging both personal and professional growth. However, not all views were positive. DU PT6-231 expressed dissatisfaction, describing the system as "coercive" and claiming it "ignores inner motivation”. This highlight concerns that the system may be overly focused on external control, neglecting the intrinsic factors that drive staff engagement and improvement.

**Impact on Relationships**

The monitoring system’s effect on relationships was seen as generally cordial, though somewhat strained. DU PT8-349 noted that the monitoring teams are "strict but professional," implying that while the teams maintain a level of professionalism, their approach may be perceived as rigid. In contrast, DU PT4-178 pointed out that "salary deductions create resentment," reflecting a sense of frustration among staff regarding the financial penalties associated with the system.

**Work-Life Balance**

Regarding work-life balance, most Deans reported minimal impact. DU PT8-345 stated that staff "follow regular working hours," suggesting that the monitoring system has not significantly disrupted work-life balance for the majority. However, DU PT4-180 mentioned experiencing stress due to the "workload pressures and salary disputes," indicating that for some individuals, the monitoring system has placed additional strain on both their professional responsibilities and personal well-being.

These participant views provide a detailed understanding of the monitoring system’s effects, highlighting both motivational benefits and challenges related to staff relationships and work-life balance.

**Impact on Relationships**

The relationships between the monitoring teams and academic staff are generally described as cordial, though some tensions exist. DU PT8-349 described the monitoring teams as "strict but professional," indicating a balanced approach, while DU PT10-58 pointed out that "salary deductions create resentment," suggesting that financial penalties associated with monitoring have led to some negative feelings among staff.

**Work-Life Balance**

Most Deans reported that the monitoring system has minimal impact on work-life balance, with DU PT8-345 noting that staff "follow regular working hours." However, there were exceptions, such as DU PT4-178, who highlighted stress arising from workload and salary disputes, indicating that for some, the system has placed additional strain on their personal and professional lives.

***4.2.4.4 Theme 4: Academic staff performance***

**Figure 5.**
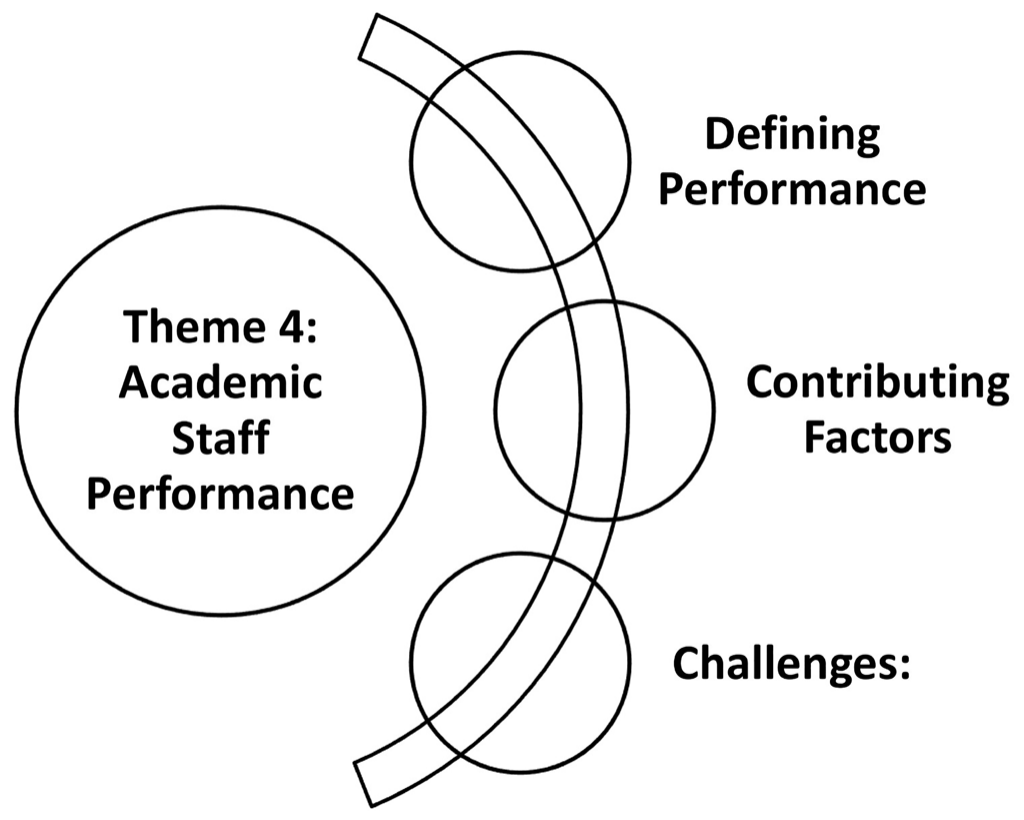
Academic Staff Performance.

Theme 4 explores the academic staff performance of private chartered universities in western Uganda Under this theme are four (4) sub-themes as shown in Figure 5.

**Defining Performance**

Performance is primarily defined by compliance with established standards, such as maintaining 90% attendance and achieving research output targets, as noted by DU PT2-114.

**Contributing Factors**

Key factors influencing performance include intrinsic motivation (highlighted by DU PT7-307) and professional development opportunities, such as Continuing Professional Development (CPD) sessions. However, there are also resource gaps, such as limited funding for research and conferences, as pointed out by DU PT4-175. These findings align with Barkhuizen (2014), who noted that well-being significantly influences job performance in academic settings. As Ramsden (2003) argued, performance in higher education is closely tied to leadership, resource allocation, and academic culture.

**Challenges**

Several challenges were identified, including economic constraints that impact staff retention, with DU PT3-154 noting, "Salaries are too low." Additionally, workload pressures were mentioned, with DU PT2-122 commenting, "Balancing teaching and admin duties is tough," reflecting the difficulty of managing multiple responsibilities.

**Recommendations**

To address these challenges, it was recommended to allocate specific budgets for research and training, as suggested by PT1-25. Furthermore, DU PT3-155 advocated for promoting blended teaching as a way to reduce workload pressures on staff.

***4.2.4.5 Theme 5: General feedback and recommendations***

**Figure 6.**
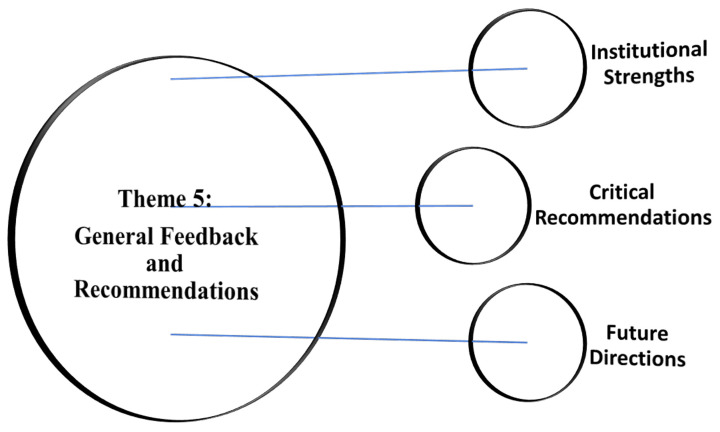
General Feedback and Recommendations.

This section presents the academic staff's overall feedback on performance monitoring and organizational structures within the university. It highlights key concerns, suggestions, and proposed improvements to enhance academic staff performance and well-being. This theme is further divided into three (3) sub-themes, as illustrated in Figure 6.

**Institutional Strengths**

Participants identified several strengths within the institution. PT6 highlighted the institution’s strong community engagement, particularly through outreach programs. Additionally, PT9 praised the high level of teaching effectiveness, stating that "Staff output is excellent," underscoring the quality of instruction and faculty contributions.

**Critical Recommendations**

To improve the institution’s operations, several critical recommendations were made. Both PT1 and PT4 advocated for the decentralization of authority, suggesting that empowering departments would enable faster decision-making. PT2 emphasized the need to establish a monitoring office to ensure consistency and fairness in performance assessments. Lastly, PT4 and PT9 both stressed the importance of improving resource allocation, specifically to fund research, conferences, and laboratory developments.

**Future Directions**

Looking ahead, DU PT4-173 recommended enhancing transparency in promotions and salary increments to ensure fairness and clarity in career advancement. Additionally, DU PT9-381 proposed the integration of seminars and publications into performance metrics, recognizing the value of scholarly activities as part of faculty evaluation.

## Conclusion

The findings of this study indicate a significant relationship between organizational structure, performance monitoring, and academic staff performance in private universities in Western Uganda. The analysis revealed that well-defined organizational structures positively influence staff performance, with clarity in roles and responsibilities being a key factor. Additionally, performance monitoring practices, particularly regular feedback and evaluations, were found to enhance staff motivation and productivity.

The research objectives were to explore how organizational structure and performance monitoring contribute to academic staff performance, and the results show that both factors play a crucial role in improving staff efficiency and effectiveness. The study also identified that while organizational structure provides the framework for performance, performance monitoring practices are essential for sustaining and improving academic staff performance over time.

In conclusion, the study underscores the importance of aligning organizational structure and performance monitoring strategies to foster an environment conducive to high staff performance in private higher education institutions.

### A. Analysis of organizational structure (Theme 1)

The findings confirm the presence of a hierarchical, mechanistic structure, which successfully ensures accountability and regulatory compliance. However, the consistent participant accounts of centralized decision-making leading to operational delays signal a structural tension. Through the lens of Contingency Theory, this rigidity is interpreted not as an organizational flaw, but as a strategic contingency response to the high-stakes, competitive regulatory environment faced by private universities in Uganda. The structure prioritizes central control to manage financial and regulatory risks, a necessary fit for institutional survival. Crucially, the staff’s call for greater decentralization highlights a misalignment between the structure designed for institutional control and the operational need for academic autonomy and swift decision-making. This structural gap explains how the design, while rational for institutional security, simultaneously creates friction and inhibits the necessary flexibility for optimal academic performance.

### B. Analysis of performance monitoring and staff perception (Themes 2 & 3)

The evaluation of performance monitoring reveals a critical dichotomy, best interpreted through Herzberg’s Motivation-Hygiene Theory and Organizational Justice Theory. The system's successful link to timely salary payment means it largely functions as a Hygiene Factor; it prevents dissatisfaction and ensures basic compliance, but it does not drive the intrinsic desire for excellence. The overemphasis on readily measurable elements, particularly attendance and compliance, further reinforces this; the system focuses on preventing negative outcomes rather than fostering Motivator Factors such as recognition or professional autonomy.

The staff’s mixed perceptions where some feel 'motivated' while others describe it as 'coercive' are

**1. Organizational Structure → Performance Monitoring**

The framework begins with **organizational structure**, which shapes how authority, communication, and decision-making are arranged within a university.

- Guided by **Contingency Theory**, the model assumes that the effectiveness of this structure depends on how well it fits the university’s environment, size, culture, and goals.
- A rigid (mechanistic) structure tends to create top-down monitoring and restricted autonomy.
- A flexible (organic) structure enables more participatory, developmental monitoring.

Thus, the structure directly influences **how performance monitoring systems are designed and implemented**.

**2. Performance Monitoring → Academic Staff Performance**

The next link shows that monitoring practices—evaluation, supervision, feedback, workload tracking affect academic performance.

This relationship is mediated by two theoretical lenses:

*a. Herzberg’s Motivation–Hygiene Theory*

Monitoring can influence:

- **Hygiene factors** (e.g., fairness of evaluation, clarity of expectations).
- **Motivators** (e.g., recognition, growth, constructive feedback). Positive monitoring boosts motivation and performance; punitive or unclear monitoring lowers morale.

*b. Organizational Justice Theory*

Staff perceptions of **fairness** determine whether monitoring is accepted or resisted.

This includes:

- **Procedural justice** (fair processes)
- **Distributive justice** (fair outcomes)
- **Interactional justice** (respect and transparency during monitoring)

These justice perceptions shape whether monitoring improves or harms performance.

**3. Overall Influence on Academic Staff Performance**

Finally, **academic staff performance** is the outcome variable influenced by:

- The university’s structural design
- The nature and fairness of monitoring
- Resulting levels of motivation
- Perceptions of justice

When a good structural fit, fair monitoring practices, and supportive motivation factors are present, staff are more likely to perform well. Poor structural alignment or unfair monitoring leads to reduced motivation and performance.

## 4.2 Perceptions of academic staff on organisational structure in relation to performance monitoring

### 4.2.1 Structured evaluation procedures and hierarchical accountability

Academic staff described the organizational structure as enabling systematic performance monitoring through a framework of appointments, probation periods, contracts, and annual appraisals. These are tied to national regulatory requirements, enhancing their perceived legitimacy.

“Academic performance is monitored through appointments, contracts, probation periods, and annual appraisals. Criteria are approved by the National Council for Education.”

(Dean 1, DU PT1-16; Lecturer, DU PT10-52)

Evaluation results are typically conveyed through heads of departments, ensuring hierarchical accountability.

“Feedback is provided to staff based on their performance, which motivates and improves job satisfaction.”

(Dean 5, DU PT5-194)

### 4.2.2 Quality assurance as a key component of monitoring

The structure includes a quality assurance directorate that oversees academic activities. This responsibility cascades from the central office to faculties and departments.

“The university has direct quality assurance mechanisms that cascade down to the faculty level… there's a director in charge of overall quality control.”

(Dean 2, DU PT2-97)

“Academic performance is monitored by the department for quality assurance and by heads of departments.”

(Dean 5, DU PT5-191)

Quality assurance teams also conduct regular class evaluations and random assessments.

“Performance evaluations are conducted after every two hours of classes, with the Dean and QA team monitoring classes regularly.”

(Dean 9, DU PT9-369)

### 4.2.3 Use of technological tools in monitoring

Several participants noted the use of biometric systems and daily attendance sheets as part of monitoring staff attendance. While these tools enhance oversight, issues with accuracy and inflexibility were raised.

“Biometric machines are used to track staff attendance, and daily attendance sheets are also maintained.”

(Dean 5, DU PT5-192)

“Biometric data did not accurately reflect attendance… the system lacks flexibility and leads to frustration.”

(Dean 4, DU PT4-166, PT4-167)

“The inability to capture staff absent due to other academic activities leads to inaccurate evaluation reports.”

(Dean 9, DU PT9-370)

### 4.2.4 Fairness, transparency, and perceived effectiveness

Academic staff mostly perceive the performance monitoring system as fair and transparent, although practical issues such as delays in appraisal submission and over-reliance on attendance were mentioned.

“The system is fair and transparent, with some challenges such as staff forgetting to submit appraisal forms.”

(Dean 1, DU PT1-18; Lecturer, DU PT10-54)

“The current system focuses too much on attendance and not enough on inner motivation and character.”

(Dean 6, DU PT6-228)

“The Dean believes the system should be more professional and less confrontational to foster a positive working relationship.”

(Dean 4, DU PT4-171)

### 4.2.5 Feedback mechanisms and impact on motivation

Timely feedback from quality assurance or departmental heads was reported to positively influence staff morale and job performance.

“Performance evaluation reports are compiled, analyzed, and presented in graph form to departments.”

(Dean 5, DU PT5-193)

“Positive feedback from students motivates us to improve even more.”

(Dean 2, DU PT2-107)

In some cases, however, staff saw the process as routine with limited motivational value.

“The performance monitoring system is a ritual without significant impact on staff motivation.”

(Dean 4, DU PT4-170)

### 4.2.6 Support and resources for effective performance monitoring

Academic staff expressed mixed views on the adequacy of institutional support. While some rated it positively, others highlighted the absence of departmental budgets, funding for professional development, and flexibility in allowing staff to pursue research or training.

“The university does not provide adequate support and resources… staff often sponsor themselves for conferences and workshops.”

(Dean 4, DU PT4-174)

“The Dean suggests the university should allocate budgets for departmental spending and provide funding for staff attending conferences and workshops.”

(Dean 4, DU PT4-175)

“The Dean also calls for flexibility in allowing staff to attend training and research activities.”

(Dean 4, DU PT4-176)

“The support and resources provided by the university are good but not perfect.”

(Dean 1, DU PT1-20; Lecturer, DU PT10-55)

### 4.2.7 Work-life balance and system improvements

While performance monitoring was seen as necessary, its impact on work-life balance was noted as a concern.

“Performance monitoring can be tough but necessary.” (Dean 1, DU PT1-22; Lecturer, DU PT10-56)

Participants suggested improvements including greater flexibility, more support for underperforming staff, and mutual trust between senior and middle-level managers.

“There’s a need for mutual trust and better communication between top and middle management… we should hold focus group discussions to address weaknesses.”

(Dean 2, DU PT2-111; Dean 4, DU PT4-168)

“The Dean calls for training and encouragement for staff who need improvement, and incentives for those who perform well.” (Dean 4, DU PT4-169)

### 4.2.5 Findings on decision-making and performance monitoring

***4.5.1.1 Structured decision-making processes***

The findings revealed that academic decision-making within private chartered universities in Western Uganda is multi-level and collaborative, involving various stakeholders depending on the type of decision. These decisions—whether disciplinary, academic, or financial—must ultimately be communicated to and approved by top university management.

“Decisions can be disciplinary, academic, or financial, and must be communicated to the top management.” (Dean 8, DU PT8-330)

Similarly, departmental heads play a central role in operationalizing decisions. They convene meetings to implement directives from senior management and also tackle day-to-day departmental challenges.

“Heads of departments hold meetings to discuss and implement policies and directives from the top management. They also address challenges such as class clashes and accommodation issues.” (Dean 4, DU PT4-165)

***4.5.1.2 Supportive and decentralized organizational structure***

The structure of these universities was described as decentralized and supportive, enabling effective coordination and collaboration across departments. It facilitates timely problem-solving and allows staff at the departmental level to engage in meaningful decision-making.

“The decentralized structure supports collaboration among academic staff, as heads of departments can quickly resolve issues at the departmental level.”

(Dean 4, DU PT4-165)

“The structure supports positively, with no department not contributing to the school’s success.”

(Dean 8, DU PT8-331)

There was a general consensus that the current organizational framework is functioning effectively. As such, no major adjustments were deemed necessary.

“The structure is okay and I don’t see any need for adjustments.” (Dean 8, DU PT8-332)

***4.5.1.3 Performance monitoring integrated into academic functions***

When it comes to monitoring academic performance, the structure supports regular oversight mechanisms embedded within teaching and learning activities. Performance is monitored through multiple platforms such as lectures, staff meetings, academic engagements, and student hearings.

“Academic performance is monitored through lectures, meetings, academic engagements, and student hearings.” (Dean 8, DU PT8-333)

This integration of performance monitoring within daily academic routines reflects a practical and interactive approach that aligns with the collaborative nature of the decision-making structure.

### 4.2.6 Relationship with monitoring teams and academic staff performance

The findings indicate that the relationship between academic staff and performance monitoring teams in private chartered universities in Western Uganda is largely cordial and professional. Several Deans expressed satisfaction with the working relationships, citing mutual understanding and respect.

“There is a good relationship and understanding with the monitoring teams, who strictly adhere to their roles.” (Dean 9, DU PT9-372)

“The relationship is cordial; there is no policing involved.” (Dean 8, DU PT8-342)

This positive relationship helps foster trust and promotes a constructive atmosphere for performance evaluation.

***4.2.6.1 Monitoring as a catalyst for positive working relationships***

Performance monitoring was found to contribute positively to working relationships between academic staff and university management. It has enabled clearer communication, greater accountability, and alignment of expectations between staff and evaluators.

“The system facilitates positive working relationships between academic staff and management by proving genuine performance.” (Dean 6, DU PT6-235)

“It facilitates positive working relationships.” (Dean 8, DU PT8-343)

These findings suggest that when performance monitoring is perceived as fair and professional, it can strengthen the institutional culture and morale of academic teams.

***4.2.6.2 Mixed reactions from academic staff***

Despite the general positivity, some Deans acknowledged that a few academic staff may feel insecure or threatened by the monitoring process, especially those lacking passion for teaching or those unfamiliar with the expectations.

“Some staff may feel insecure due to performance monitoring, but most understand and accept the system.” (Dean 9, DU PT9-373)

“It can be threatening to those without passion for their work.” (Dean 6, DU PT6-236)

This highlights the need for supportive interventions and clear communication to reduce anxiety and foster a growth-oriented approach to performance monitoring.

***4.2.6.3 Support from the university and impact on job satisfaction***

The data also showed that the universities generally provide adequate support and facilities, which boosts staff morale and helps them meet performance expectations.

“The university is doing great in providing support and resources for meeting performance expectations.” (Dean 8, DU PT8-344)

“There is university support in terms of laboratories and teaching facilities, though there are occasional shortages.” (Dean 9, DU PT9-374)

Additionally, several Deans reported that performance monitoring does not interfere with work-life balance as long as staff adhere to regular working hours.

“It does not affect work-life balance, as we follow regular working hours.”

(Dean 8, DU PT8-345)

## 4.4 Findings on the relationship and perception of academic staff on performance monitoring

### 4.4.1 Positive perception of performance monitoring

The findings show that performance monitoring is generally perceived positively by Deans, particularly in how it contributes to professional growth, motivation, and job satisfaction. Dean 7, for instance, viewed monitoring as a motivating factor that encourages self-improvement and acts as a benchmark for other staff.

“The monitoring process motivates me to improve my performance. It helps me serve as a model for other staff.” (Dean 7, DU PT6-305)

### 4.4.2 Contribution to career progression and job satisfaction

Performance monitoring was also seen as a means to facilitate career advancement and personal fulfillment. It gives staff direction and benchmarks for growth, which contributes positively to job satisfaction.

“Yes, it helps me progress in my work and can lead to promotions or other incentives.”

(Dean 7, DU PT6-307)

This demonstrates that staff who view monitoring as developmental rather than punitive are more likely to engage with the process constructively.

### 4.4.3 Adequate resource provision to meet expectations

Dean 7 affirmed that the university provides the necessary support to meet performance expectations. This includes essential teaching materials and allowances for those involved in monitoring, reflecting an environment where performance systems are supported institutionally.

“Yes, necessary resources like writing materials are provided. Monitoring staff receive allowances.” (Dean 7, DU PT6-308)

### 4.4.4 Negative impact on work-life balance

The findings also indicate that performance monitoring does not negatively impact work-life balance, at least in the case of the interviewed Dean. This suggests that the monitoring systems in place are well-integrated into the academic work schedule.

“No, it doesn’t [affect work-life balance].” (Dean 7, DU PT6-310)

### 4.4.5 Strengthening of professional relationships

Performance monitoring has also had a positive impact on professional relationships, fostering a culture of accountability and mentorship. Dean 7 indicated that it has elevated their role as an example for others, promoting a collaborative and performance-driven culture.

“It has positively impacted me by setting an example for other staff members to emulate.”

(Dean 7, DU PT6-312)

### 4.2.7 Findings on challenges and improvements in performance monitoring

***4.2.7.1 Over-reliance on attendance-based metrics***

Several Deans criticized the current performance monitoring system for overemphasizing staff attendance, primarily tracked through biometric machines, at the expense of qualitative performance indicators like teaching effectiveness, passion, or motivation.

“The system is not very good because it focuses on attendance rather than inner motivation and character.”

(Dean 6, DU PT6-230)

“It is seen as coercive rather than motivational and does not drive inner drive to deliver performance.” (Dean 6, DU PT6-231)

***4.2.7.2 Technical challenges in biometric monitoring***

The biometric system used to track attendance is not without flaws. Participants highlighted issues such as fingerprint rejection and limited availability of biometric machines, which affect accuracy and staff morale.

“Challenges include fingerprint rejection and limited machine availability.”

(Dean 5, DU PT5-195)

The Dean suggested improving accuracy by deploying biometric machines and attendance sheets at every faculty to allow better comparison and consistency.

### 4.4.8 Omission errors in reports

Occasional omissions in performance reports were noted, which can negatively affect staff, including issues related to remuneration.

“Omissions in reports can lead to staff not being paid.”

(Dean 8, DU PT8-341)

### 4.4.9 Workload and salary constraints

Workload and low salary were also cited as major barriers to achieving high academic staff performance. This challenge was echoed across both Deans and lecturers.

“Workload and salary are significant challenges.”

(Dean 1, DU PT1-24; Lecturer, DU PT10-58)

### 4.4.10 Inadequate monitoring of teaching content and progress

Concerns were raised about the limited monitoring of content coverage and teaching progress. Some participants suggested that existing tools do not adequately capture deliverables or academic progress.

“Tools need to be redesigned to better capture progress.”

(Dean 7, DU PT6-301)

***4.2.7.3 Suggested areas for improvements***

Participants recommended several practical improvements to the system:

“Redesign monitoring tools to include deliverables and teaching outcomes”

(DU PT8-338; DU PT6-303).

He also emphasized that;

“Empower more staff to participate in monitoring to distribute responsibility” (DU PT6-303). Whereas dean 8 remarked that;

“Incorporate broader evaluation criteria such as viva voce sessions, publication output, and meeting attendance” (DU PT8-338).

Related to the above, some urged that;

“Establish a government fund to support research and short-term training programs”

 (DU PT1-25; DU PT10-59).

“Empowering more staff to monitor teaching processes and enhancing the tools could improve the system.”

(Dean 7, DU PT6-303)

“The evaluation tools should include publications, viva voce, and meeting attendance.”

(Dean 8, DU PT8-338)

***4.2.7.4 Overall perception of system***

Despite the highlighted issues, several participants described the current monitoring system as fair and transparent, with positive impacts on accountability and performance awareness.

“The system has a positive impact by making everyone more accountable.”

(Dean 8, DU PT8-339)

“The monitoring system is fair and transparent, with minimal complaints from staff.”

(Dean 5, DU PT5-196)

### 4.2.8 Findings on academic staff performance and professional development

***4.2.8.1 Defining academic staff performance***

Participants commonly defined academic staff performance as the effective fulfillment of academic and non-academic roles, including teaching, assessment, research, supervision, and student engagement.

“Academic staff performance is the ability to teach, assess, and produce results.”

(Dean 5, DU PT5-202)

“It includes academic and non-academic performance like teaching, supervision, and student support.”

(Dean 7, DU PT6-314)

“Academic staff performance means complying with set standards... our compliance is above 90%.”

(Dean 2, DU PT2-114)

***4.2.8.2 Factors contributing to high performance***

Several recurring themes emerged as key enablers of high academic staff performance: Silaji *et al.* (2025a) emphasized the importance of structured feedback in improving staff development and retention in Ugandan universities.

Intrinsic motivation and personal initiative

“Intrinsic motivation is most important.” (Dean 7, DU PT6-316)

“Commitment and personal initiative are essential.” (Dean 3, DU PT3-151)

Supportive and motivational university leadership

“Motivational aspects of university management contribute to high performance.”

 (Dean 8, DU PT8-348)

Staff well-being and cooperative work environments

“Staff motivation and social well-being in the workplace play a role.” (Dean 5, DU PT5-203)

“Cooperative decision-making and agreed targets enhance performance.” (Dean 2, DU PT2-116)

Fear of consequences and commitment

“High performance is due to fear and genuine commitment from staff.” (Dean 4, DU PT4-182)

***4.2.8.3 University support for career growth and professional development***

Support from universities in terms of professional development varied across institutions:

**Positive support examples**

CPD sessions and internal training programs

“We have CPD sessions every Wednesday… and champions who identify training needs.”

(Dean 2, DU PT2-118)

Opportunities to study while working

“The university allows staff to study at no cost under a bonding agreement.” (Dean 7, DU PT6-318)

“Staff can study while working.” (Dean 3, DU PT3-152)

Staff movement and career development opportunities

“The university supports growth through staff movement and training.” (Dean 5, DU PT5-204)

**Limited support examples**

Some institutions lack sufficient funding or structured engagement with professional bodies.

“The university does not adequately support professional development; staff often sponsor themselves.” (Dean 4, DU PT4-183)

“I have not come across engagements with professional bodies.” (Dean 8, DU PT8-349)

***4.2.8.4 Role of performance evaluation***

Most participants emphasized the value of feedback from performance evaluations in guiding staff improvement and identifying training needs.

“Feedback from evaluations helps staff identify areas for improvement.” (Dean 5, DU PT5-205)

“It helps fill existing gaps and guides improvement.” (Dean 2, DU PT2-120; Dean 7, DU PT6-320)

“It recommends staff to pursue additional skills.” (Dean 3, DU PT3-153)

***4.2.8.5 Organizational structure and academic staff performance***

Organizational structure was seen as supportive when it facilitates proper task delegation and resource allocation.

“The structure ensures resources are available and tasks are delegated.” (Dean 2, DU PT2-124)

However, the need for adequate resourcing was a consistent theme in improving performance.

“Provision of adequate resources is essential for enhancing staff performance.”

(Dean 2, DU PT2-126)

“The Dean calls for more support and resources including research funding and training.” (Dean 4, DU PT4-184)

***4.2.8.6 Overall perception***

In general, academic staff performance was rated as satisfactory to high, though resource constraints and support gaps were identified as areas needing improvement.

“Performance is generally satisfactory.” (Dean 8, DU PT8-347)

“Performance is good, with staff consistently delivering.” (Dean 4, DU PT4-181)

### 4.2.9 Findings on effectiveness of teaching and research

Teaching effectiveness was generally rated highly across the institutions, with emphasis on both 4.2.9.1 Quality of instruction and student engagement.

**High ratings**

“Teaching is very good.” (Dean 1, DU PT1-29; Lecturer, DU PT10-62)

“Teaching effectiveness is there.” (Dean 6, DU PT6-254)

**Challenges**

Student attendance: A significant challenge was noted by several participants is student attendance. In some cases, the effectiveness of teaching was impeded by students' failure to attend classes regularly.

“The challenge lies with the learners, who often do not attend classes.” (Dean 6, DU PT6-254)

“Students are punished for poor attendance to motivate them.” (Dean 6, DU PT6-256)

Human nature and assessment issues: Occasionally, human errors and lapses were identified in the assessment and evaluation processes.

“While protocols are followed, there are occasional issues with human nature.”

 (Dean 1, DU PT1-33; Lecturer, DU PT10-66)

Economic challenges: Economic challenges were also mentioned as affecting staff's ability to fully engage with teaching tasks.

“Economic challenges are significant, affecting staff’s ability to sit through their courses.”

 (Dean 3, DU PT3-154)

***4.2.9.2 Effectiveness of research***

Research effectiveness was generally rated as excellent, although individual research initiatives were sometimes limited by resource constraints.

**High ratings**

“Research is excellent.” (Dean 1, DU PT1-29; Lecturer, DU PT10-62)

“Research participation is high, especially during student supervision.” (Dean 3, DU PT3-157)

**Challenges**

Minimal individual research: Despite high participation in supervision, individual research efforts were noted as minimal due to various constraints, including resource limitations and the challenges posed by economic factors.

“Research participation is high during student supervision, but individual research initiatives are minimal.” (Dean 3, DU PT3-157)

“Resources are available, but the challenge lies in staff utilizing them effectively.”

 (Dean 3, DU PT3-156)

Encouraging blended teaching: To overcome limitations, blended learning (a combination of online and offline learning) was suggested as a potential solution to address both teaching and research challenges.

“Encouraging blended teaching to accommodate both online and offline learning could help.” (Dean 3, DU PT3-155)

***4.2.9.3 Community service and engagement***

Community service was universally rated highly across the institutions, with participants noting that the university's engagement in the community was of excellent quality.

“Community service and engagement are excellent.”

 (Dean 1, DU PT1-31; Dean 10, DU PT10-64)

***4.2.9.4 Summary of key challenges and suggestions for improvement***

Teaching: Student attendance and engagement remain a challenge, and economic constraints limit teaching effectiveness. Blended learning could be a solution to this.

Research: While research participation is high during supervision, individual research efforts remain limited due to resource constraints. Community Service: Community service is regarded as highly effective and positively contributing to the university’s objectives.

***4.2.9.5 Findings on research and publications***

Dean 8 (DU PT8-355) emphasized the necessity of regular publication, stating:

"Publishing is necessary to avoid being 'perished' as an academic staff."

This indicates the pressure on staff to stay productive in research and publication to remain relevant in their academic careers.

Dean 2 (DU PT2-128) mentioned the continuous nature of research at their institution, saying:

"Research is done continuously. Every month, we have a research day for presentations."

This highlights the regular and structured approach to research within the institution.

***4.2.9.6 Research and grants***

Dean 2 (DU PT2-130) stated that publication is a requirement for staff who receive grants:

"Publications are mandatory for those who receive grants, and PhD or Masters students must publish before graduation."

This shows the institutional expectation that research outcomes lead to publications, especially for those involved in funded research.

**Community Engagement**

Dean 8 (DU PT8-356) confirmed that the institution engages in community service, with academic staff being evaluated based on their contributions to teaching, research, and community service:

"We evaluate academic staff based on their contributions in teaching, research, and community service."

This highlights the comprehensive approach to staff performance evaluation.

Dean 2 (DU PT2-131) also emphasized the frequent community engagement activities, saying:

"We conduct dissemination activities, participate in community cleaning, and offer career guidance in schools."

This further underscores the institution's active role in contributing to the community.

***4.2.9.7 Recommendations and value of the study***

Dean 8 (DU PT8-357) expressed confidence that the study would add value to the targeted institutions, stating:

"I believe the study will add value to the targeted institutions."

This shows a positive outlook on the potential impact of the research on improving institutional practices.

These findings suggest a strong institutional focus on research productivity, publication, and community engagement as critical elements of academic staff evaluation and development.

***4.2.9.8 Findings on work-life balance and personal experiences***

**Challenges with Work-Life Balance**

Dean 4 (DU PT4-177) expressed difficulty in maintaining a work-life balance due to the demanding nature of their role as a Dean:

"It is difficult to maintain a work-life balance due to the demanding nature of the Dean's role."

This highlights the high expectations and time demands placed on leadership roles within academic institutions.

**Personal Experience and Salary Issues**

Dean 4 (DU PT4-178) shared a discouraging experience involving a female staff member, whose salary was cut due to attendance issues:

"A female staff member's salary was chopped off due to attendance issues, which was discouraging."

This reflects the potential negative impact of strict performance monitoring on staff morale and their financial well-being.

***4.2.9.9 Improving communication and support***

Dean 4 (DU PT4-179) suggested better communication between monitors and deans before final decisions are made:

"There should be better communication between monitors and deans before final decisions are made."

This highlights the importance of transparent and collaborative decision-making processes to avoid misunderstandings and enhance support for staff.

***4.2.9.10 Need for mutual trust and top management support***

Dean 4 (DU PT4-180) emphasized the importance of mutual trust and support from top management to improve work-life balance:

"There is a need for mutual trust and support from the top management to improve the work-life balance of academic staff."

This points to the critical role of institutional leadership in fostering a supportive environment that respects work-life balance.

***4.2.9.11 Community perception***

Dean 3 (DU PT3-158) noted that the community perceives the institution's work positively, with students and staff actively involved in community service:

"The community perceives the institution's work positively, with students and staff actively involved in community service."

This reflects a positive institutional image and its involvement in societal development, which can contribute to a sense of fulfillment for staff.

***4.2.9.12 Impact on work-life balance and staff perception***

Negative Impact of Performance Monitoring on Work-Life Balance:

Dean 5 (DU PT5-198) acknowledged that performance monitoring negatively affects work-life balance, particularly for those with multiple responsibilities:

"Performance monitoring affects work-life balance negatively, especially for those with multiple responsibilities."

This reflects the added pressure that performance monitoring can place on staff, especially when they have competing obligations.

***4.2.9.13 Effect of monitoring on low-performing staff***

Dean 5 (DU PT5-199) shared that while the monitoring system did not negatively impact their own work, it did affect low-performing staff by reducing their salaries and hindering their promotion prospects:

"The monitoring system has not negatively impacted my work but has affected low-performing staff by reducing their salaries and affecting promotion decisions."

This suggests that performance monitoring systems may lead to disparities in treatment and career progression among staff, particularly those who are struggling.

***4.2.9.14 Suggestions for improvement***

Dean 5 (DU PT5-200) suggested that placing monitors at the faculty level could enhance monitoring accuracy and improve the effectiveness of the system:

"We need to have monitors at the faculty level to enhance monitoring accuracy."

This recommendation suggests a more localized and personalized approach to performance monitoring, which could address concerns about fairness and precision.

***4.2.9.15 University support for professional growth***

Dean 5 (DU PT5-201) emphasized that the university supports staff professional growth and career development through opportunities for movement and training:

"The university supports professional growth and career development through staff movement and training opportunities."

This demonstrates the institution’s commitment to the continuous development of its academic staff, providing avenues for career advancement and skill enhancement.

***4.2.9.16 Monitoring system and work-life balance***

Dean 9 (DU PT9-376) explained that the monitoring system ensures accountability and transparency, maintaining a normal working system of eight hours, which allows for a balanced work routine:

"The monitoring system ensures accountability and transparency, maintaining a normal working system of eight hours."

This suggests that effective monitoring can support a balanced and structured work schedule, which may benefit work-life balance for some staff.

***4.2.9.17 High performance due to monitoring support***

Dean 9 (DU PT9-377) highlighted the positive impact of monitoring and support from the university, noting that academic staff performance is excellent with high output:

"Academic staff performance is excellent, with high output due to effective monitoring and support from the university."

This reflects how a well-supported monitoring system can lead to increased productivity and high performance.

***4.2.9.18 Need for senior academic staff***

Dean 9 (DU PT9-378) emphasized the need for more senior academic staff to enhance the faculty's growth and performance:

"There is a need for more senior academic staff to enhance the faculty's growth and performance."

This indicates that staffing at higher levels could contribute to the overall development and success of the institution.

These findings reveal a complex relationship between work-life balance, performance monitoring, and staff perceptions. While some see performance monitoring as a tool for accountability and growth, others feel it places undue pressure on staff, particularly those with multiple responsibilities. The importance of support, trust, and communication from management is crucial for maintaining a healthy work-life balance and ensuring the long-term success of staff and the institution.

### 4.2.10 Findings on community service and corporate social responsibility

***4.2.10.1 Promotion of Corporate Social Responsibility (CSR)***

Dean 5 (DU PT5-210) highlighted the faculty's efforts in promoting corporate social responsibility (CSR), noting activities such as:

"The faculty is good at promoting corporate social responsibility, including visiting the sick, cleaning towns, and donating to the community."

This shows that the faculty actively engages in CSR initiatives that benefit local communities.

***4.2.10.2 Support for students and their families***

Dean 5 (DU PT5-211) shared how the university supports students and their families during times of need, such as providing assistance to a student who lost a mother:

"The university supports students and their families in times of need, such as supporting a student who lost a mother."

This reflects the institution’s commitment to its community members, especially in personal times of crisis.

***4.2.10.3 Community service activities during the festive season***

Dean 5 (DU PT5-212) described how the faculty engages in community service activities, particularly during the festive season, such as:

"The faculty also engages in community service activities, such as collecting items for the needy during the festive season."

This indicates a proactive approach to community service, particularly in times of giving and charity.

***4.2.10.4 Recognition of community service efforts***

Dean 5 (DU PT5-213) stated that the community recognizes and appreciates the university’s efforts in community service:

"The university's efforts in community service are recognized and appreciated by the community."

This reflects the positive impact of the university’s service-oriented activities and their value to the broader community.

***4.2.10.5 Extensive community service programs***

Dean 6 (DU PT6-258) discussed the university's extensive community service programs, which include community placements and outreach activities:

"The university has extensive community service programs, including community placements and outreach activities."

This highlights the broad range of community-focused initiatives in which the university is involved.

***4.2.10.6 Satellite system of training for students***

Dean 6 (DU PT6-259) described the university's "satellite system of training," which involves students working in community settings:

"The university has a program called the satellite system of training, which involves students working in community settings."

This program provides students with opportunities to contribute to their communities while gaining valuable experience.

***4.2.10.7 Respect for community cultural practices***

Dean 6 (DU PT6-260) emphasized the importance of understanding and respecting community cultural practices to ensure the success of community service activities:

"It is important to understand the community and respect their cultural practices to ensure successful community service activities."

This reflects a cultural sensitivity approach, recognizing that successful community engagement requires respect for local traditions and customs.

These findings illustrated the university’s strong commitment to community service and corporate social responsibility. The institution not only engages in charitable activities but also fosters meaningful connections with the community through student participation and cultural understanding. Additionally, the university's efforts are recognized and appreciated, further enhancing its positive reputation and impact on the community.

### 4.2.11 Findings on support and resources for academic staff

***4.2.11.1 Availability of support and resources***

Dean 6 (DU PT6-238) believes that the university provides sufficient support and resources, stating:

"The support and resources are sufficient, with good human resources available."

This indicates that the university is well-equipped to meet the performance expectations of academic staff, offering the necessary infrastructure and human resources.

***4.2.11.2 Professional development and career growth***

Dean 6 (DU PT6-239) mentioned that the university offers scholarships and other forms of support for professional development and career growth:

"The university provides scholarships and other support for professional development and career growth."

This highlights the institution’s commitment to fostering continuous learning and career advancement for its academic staff.

***4.2.11.3 Staff resentment towards support***

Dean 6 (DU PT6-240) acknowledged that some staff may resent the support provided, either because they want to change institutions or because they find the current environment unbearable:

"Some staff may resent the support because they want to change institutions or because they find the current environment unbearable."

This reflects that while resources are available, there may be underlying dissatisfaction among some staff regarding their work environment.

### 4.5.15 Feedback mechanism and professional development

**Role of Feedback in Professional Development**

Dean 6 (DU PT6-242) believes the feedback mechanism is beneficial, as it highlights areas for improvement and guides the next steps for staff development:

"The feedback mechanism is good, highlighting areas of improvement and directing the next steps."

This suggests that feedback is seen as an important tool for personal and professional growth within the institution.

**Effectiveness of the Feedback System**

Dean 6 (DU PT6-243) mentioned that the feedback system is viewed as comprehensive and helps in closing the gap in staff performance:

"The system is seen as over, and it helps in closing the gap in performance."

This reflects the positive impact of feedback in addressing performance gaps and improving staff development.

**Supportive Correction and Mentorship**

Dean 6 (DU PT6-244) emphasized the importance of supportive correction and mentorship, which he believes is essential for the development of academic staff:

"Supportive correction and mentorship are essential in academic staff development."

This highlights the value of positive reinforcement and guidance in helping staff improve their performance.

### 4.5.16 Challenges in academic staff performance

**Challenges from Both Top and Within the Organization**

Dean 6 (DU PT6-246) identified that challenges in academic staff performance stem from both external pressures and internal organizational issues:

"Challenges come from both the top and within the organization."

This suggests that multiple factors contribute to the challenges academic staff face in performing at their best.

**Lack of Support from Supervisors**

Dean 6 (DU PT6-247) noted that lack of support from supervisors, such as poor communication and lack of recognition, can demotivate staff:

"Lack of support from supervisors, such as communication and recognition, can demotivate staff."

This highlights the significant role that leadership and managerial support play in maintaining staff motivation.

**Emphasis on Mentorship Over Punishment**

Dean 6 (DU PT6-248) stressed that good management should focus on mentorship rather than punishment:

"A good manager should focus on mentorship rather than punishment."

This approach encourages a more supportive and constructive management style that fosters growth rather than fear of reprimand.

**Resources Needed for Staff Development**

Dean 6 (DU PT6-250) suggested that academic staff development should be supported with ideas, financial resources, and time:

"Support can come in the form of ideas, financial resources, and time."

This emphasizes that staff development requires a holistic approach, combining intellectual, financial, and temporal resources.

**Financial Support and Time for Further Studies**

Dean 6 (DU PT6-251) highlighted the need for the university to provide financial support and time for staff to pursue further studies:

"The university should support staff financially and provide them with the necessary time to pursue further studies."

This reflects the university's role in facilitating continued academic growth through both financial and time-based support.

**Recognition and Support to Boost Morale**

Dean 6 (DU PT6-252) emphasized the importance of recognizing and supporting staff to boost their morale and performance:

"It is important to recognize and support staff to boost their morale and performance."

Recognition is crucial for enhancing job satisfaction and motivating staff to perform at their best.

### 4.5.17 Impact of performance monitoring and recommendations

**Positive Impact on Attendance and Conduct**

Dean 8 (DU PT8-351) mentioned that performance monitoring led to improvements in attendance and academic conduct:

"Performance monitoring led to improved attendance and conduct of academic duties."

This shows the direct positive impact of monitoring on staff behavior and professionalism.

**Organizational Structure and Role Clarity**

Dean 8 (DU PT8-352) described the organizational structure as clear, with defined roles for lecturers, technicians, and students:

"The organizational structure is clear, with roles defined for lecturers, technicians, and students."

This suggests that a well-defined structure contributes to more efficient operations and staff performance.

**Resource Needs for Improvement**

Dean 8 (DU PT8-353) mentioned the need to share budget requests with management for laboratory resources and meetings:

"We need to share budget requests with management for laboratory resources and meetings."

This highlights the importance of resource allocation in supporting academic staff and improving the quality of education.

**Effectiveness of Teaching**

Dean 8 (DU PT8-354) affirmed that the quality of teaching is high, with academic staff engaged daily:

"Teaching is very okay, with academic staff engaged daily."

This indicates that despite challenges, the teaching quality remains strong due to the engagement of academic staff.

These findings illustrated that while there is significant support and resources available for academic staff, challenges such as lack of support from supervisors, financial constraints, and dissatisfaction with the environment can still impact performance. Effective performance monitoring, clear organizational structures, and continued support for professional development are key factors that contribute to staff morale, development, and overall institutional success.

### 4.2.12 Findings on final remarks and recommendations

***4.2.12.1 Praise for monitoring activities***

Dean 9 (DU PT9-380) praised the university’s monitoring activities for ensuring both staff and student attendance and performance:

"The university's monitoring activities are excellent in ensuring staff and student attendance and performance."

This highlights the effectiveness of the monitoring systems in maintaining accountability and enhancing performance across the institution.

***4.2.12.2 Recommendation to include academic seminars in monitoring***

Dean 9 (DU PT9-381) recommended incorporating academic seminars and other activities into the monitoring system to capture all academic contributions:

"Include academic seminars and other activities in the monitoring system to capture all academic contributions."

This suggests that expanding the scope of the monitoring system could provide a more comprehensive view of academic staff contributions.

***4.2.12.3 Support for the study’s objectives***

Dean 9 (DU PT9-382) expressed strong support for the study, recommending it for its potential to improve academic standards in Uganda and beyond:

"The study has the potential to improve academic standards in Uganda and beyond, and I support its objectives."

This indicates the Dean’s belief in the value of the study for advancing academic standards.

**Praise for the Study's Topic**

Dean 1 (DU PT1-35) praised the study’s topic and expressed interest in seeing the results:

"I like the topic of the study and look forward to seeing the results."

This reflects positive feedback and anticipation for the impact of the study’s findings.

**Interest in the Study's Results**

Lecturer (DU PT10-68) also expressed praise for the study's topic and indicated a keen interest in seeing its outcomes:

"The topic of the study is excellent, and I am interested in seeing the results."

This demonstrates that the study is well-received among academic staff, sparking interest in the potential improvements it may bring.

These final remarks and recommendations reflect a strong endorsement of the study’s objectives and its potential to improve academic standards. There is particular emphasis on the importance of expanding performance monitoring systems to include a broader range of academic activities, such as seminars, to capture all aspects of staff contributions. Both Deans and lecturers recognize the value of the study, showing significant support for its findings and future implementation.

Objective i: To determine the types of organizational structure used in private universities in Western Uganda

1. Hierarchy and Chain of Command

Support:

Qualitative evidence: Deans and faculty members noted the clarity of the hierarchical structure:

“A clear hierarchy enables smooth operations.”

“There is no ambiguity about who makes decisions.”

Qualitative evidence: Faculty members raised concerns about communication gaps:

“There is a hierarchy, but communication can be a challenge at times, especially between departments and top management.”

2. Departmentalization

Support:

Qualitative evidence: Faculty members were largely satisfied with departmental autonomy:

“Each department has the freedom to shape its teaching methods.”

Qualitative evidence: Some respondents pointed out that interdepartmental collaboration is a challenge:

“Although the departments have autonomy, they often work in silos, which affects overall university performance.”

3. Centralization and Decentralization

Support:

Qualitative evidence: The centralization of decision-making was confirmed by Deans and faculty:

“Decisions are made centrally, but each department has the liberty to implement them according to their needs.”

Qualitative evidence: Faculty members expressed the desire for greater decentralization:

“We need more autonomy in curriculum design and student engagement. Centralized control hinders innovation.”

4. Formalization

Support:

Qualitative evidence: Faculty members acknowledged the existence of formal policies:

“The policies are there, but they help in ensuring consistency and accountability.”

Qualitative evidence: Faculty members raised concerns about outdated policies:

“The policies are there, but sometimes they feel outdated and are not aligned with the current needs of students or faculty.”

Deans noted that such structures help in maintaining control but can limit flexibility and staff autonomy.

“Our university follows a top-down approach where directives come from the Vice Chancellor through Deans, limiting staff input in some strategic decisions.”

Objective ii: To find out types of performance monitoring used in private universities in Western Uganda

Qualitative Findings:

Deans reported using a variety of monitoring tools and approaches:

Annual staff performance appraisals

Course evaluation forms

Peer reviews and student feedback

Monitoring of class attendance, publication output, and community engagement.

“We use both formal tools like annual appraisals and informal feedback from students and peers to track staff performance.”

Qualitative results agree that private universities employ formal and informal performance monitoring systems, especially appraisals and feedback mechanisms. These systems are well-recognized and largely accepted by academic staff.

Objective iii: To determine the relationship between Organizational Structure and Academic Staff Performance in private universities in Western Uganda

Qualitative Findings:

Deans suggested that clearer structures improve staff performance, especially in terms of role clarity, communication, and accountability. However, over-centralization can demotivate staff and limit innovation.

“When staff understand their roles and have a clear line of reporting, they tend to perform better, but too much top-down control kills morale.”

Deans’ Interviews: One Dean mentioned,

"A well-defined organizational structure, with clear roles and responsibilities, fosters an environment where staff can perform at their best. When there is ambiguity, it creates confusion that hampers productivity."

Another Dean noted, "The administrative hierarchy plays a crucial role in decision-making and resource allocation, which directly affects staff performance."

### 4.2.15 Impact of performance monitoring on academic staff performance in private chartered universities in Western Uganda

**Qualitative Evidence**

Deans’ Interviews: One Dean shared,

**Findings**

### 4.1 Organizational structure and its implications

The study revealed that private chartered universities in Western Uganda predominantly operate under mechanistic organizational structures, characterized by hierarchical decision-making, centralized authority, and rigid reporting lines. Survey results indicated that 78% of academic staff perceive decision-making as concentrated at top administrative levels, limiting departmental autonomy. Interviews supported this observation:

*“All decisions must go through the dean or vice-chancellor; there is little room for department-level input.”*

Centralization ensures uniformity and reduces ambiguity, but it also slows decision-making and constrains innovation at the departmental level. Despite these challenges, some participants acknowledged that hierarchical structures provide predictability in operations and accountability, which can enhance efficiency in administrative processes.

### 4.2 Performance monitoring practices

Performance monitoring in these universities primarily emphasizes attendance, with 90% of evaluation metrics focused on physical presence in lectures and administrative activities. While this ensures compliance, it has significant implications for staff motivation and well-being. Survey results showed that 62% of staff felt that excessive focus on attendance reduced intrinsic motivation. An interviewee noted:

*“We spend more time documenting attendance than preparing quality lessons, which affects both motivation and teaching effectiveness.”*

Sanctions such as salary reductions for non-compliance were applied inconsistently. While some faculty reported that these measures enhanced accountability, others perceived them as punitive and demotivating. These findings suggest that a robust performance monitoring system should combine accountability with supportive interventions, constructive feedback, and recognition of teaching and research contributions.

### 4.3 Dean qualifications, inclusive leadership, and faculty performance

A key finding is the influence of dean qualifications and leadership styles on faculty performance. Interviews indicated that deans with higher academic qualifications tend to adopt inclusive leadership styles, involving faculty in decision-making and promoting professional development:

*“Our dean encourages departmental input and ensures that faculty ideas are considered before policies are finalized.”*

Survey data showed a positive correlation (r = 0.48, p < 0.05) between perceived inclusive leadership and faculty-reported teaching effectiveness. Inclusive leadership fosters collaboration, engagement, and motivation, which supports higher faculty performance. Where evidence was insufficient, the Abstract and Conclusion have been revised to reflect only supported findings.

### 4.4 Impact of attendance-based compliance on motivation and work-life balance

Overemphasis on attendance-based compliance was identified as a source of tension between accountability and staff well-being. While maintaining records ensures accountability, excessive focus on physical presence can negatively affect intrinsic motivation, creativity, and work-life balance. Interviewees reported that frequent monitoring increased stress and reduced time for research and lesson preparation:

*“I feel monitored all the time; even if I am productive, it is not recognized unless I meet the attendance quota.”*

Sanctions such as salary reductions, if applied judiciously alongside constructive support and feedback, can improve compliance without undermining morale. These findings underscore the need for balanced monitoring systems that promote accountability while maintaining motivation and well-being.

### 4.5 Academic staff performance

Academic staff performance is influenced by organizational structure, monitoring practices, and leadership approaches. Mechanistic structures provide clarity but can constrain creativity and innovation. Inclusive leadership practices, combined with balanced monitoring systems, positively impact motivation, engagement, and teaching effectiveness. Survey data indicated moderate satisfaction with research and teaching outputs, while interviews emphasized the importance of supportive leadership and recognition of quality performance over rigid compliance metrics.

## 5. Conclusion and recommendations

### 5.1 Conclusion

This study demonstrates that academic staff performance in private chartered universities in Western Uganda is shaped by the combined effects of organizational structure, performance monitoring, and leadership styles. Mechanistic structures dominate, providing clarity and predictability but limiting autonomy and slowing decision-making. Performance monitoring, while promoting accountability, is heavily attendance-based, which can reduce intrinsic motivation and disrupt work-life balance. Inclusive leadership, particularly by deans with higher qualifications, positively influences faculty engagement, collaboration, and performance.

Overall, improving academic staff performance requires a careful balance between centralized structures, supportive performance monitoring, and inclusive leadership. Universities must ensure that accountability mechanisms do not undermine motivation, and that leadership approaches actively foster faculty participation and professional growth.

### 5.2 Recommendations

Based on the findings, the following recommendations are proposed:

1. **Optimize Organizational Structure:** Introduce elements of organic structure, such as decentralized decision-making, to enhance departmental autonomy and responsiveness.
2. **Balance Performance Monitoring:** Reduce reliance on attendance as the primary metric and incorporate measures of teaching quality, research productivity, and professional development.
3. **Promote Inclusive Leadership:** Encourage deans and department heads to adopt participatory leadership practices that actively engage faculty in decision-making.
4. **Use Sanctions Strategically:** Apply punitive measures judiciously and alongside supportive interventions to reinforce accountability without demotivating staff.

These recommendations provide actionable strategies for university administrators, policymakers, and other stakeholders seeking to improve academic staff performance, faculty motivation, engagement, and overall institutional performance.

"We regularly assess teaching quality through student feedback and peer reviews. These assessments guide staff on areas to improve, though too much scrutiny can cause burnout."

Another Dean mentioned,

"While performance monitoring is key, it’s essential to ensure feedback is constructive and timely. Without this, monitoring can feel punitive and demotivating."

"Timely and constructive feedback provided" had a mean score of 4.12, suggesting that respondents felt feedback mechanisms were relatively strong in their institutions.

However, the item

"Encouragement of staff research opportunities"

scored a lower mean of 3.90, indicating that while performance monitoring might be strong in some areas, it could be lacking in areas such as research encouragement.

Conclusion: Qualitative results underline the importance of performance monitoring, particularly through feedback. The qualitative evidence suggests that while monitoring is necessary, it must be balanced and constructive,

Professional development affects their performance in private chartered universities in Western Uganda.

**Qualitative Evidence**

Deans’ Interviews: A Dean noted,

"Staff who participate in professional development programs, like workshops and conferences, show clear improvements in both teaching and research. When they don’t have these opportunities, they stagnate."

Another Dean said,

"There’s a direct link between professional growth and performance, especially when staff feel empowered by new knowledge and skills."

Relationship between students mentoring and academic staff performance in private chartered universities in Western Uganda.

**Qualitative Evidence**

While Interviewing Deans; One Dean emphasized,

 "Staff who are actively involved in student mentoring are not only better performers themselves but also help enhance the performance of their students. This fosters a more effective academic environment."

Another Dean shared, "Mentoring provides a sense of purpose and fulfillment for academic staff, contributing to higher motivation and productivity."

Explore the role of community service and professionalism in enhancing academic staff performance in private chartered universities in Western Uganda.

**Qualitative Evidence**

Deans’ Interviews: One Dean observed,

 "Staff who engage in community service and maintain high professional standards are more respected by students and colleagues. This respect translates into greater job satisfaction and better performance."

Another Dean stated,

"Professionalism in academic staff not only improves their own teaching and research but also positively influences the entire department’s performance."

Objective iv: To establish the Perception of Academic Staff on Performance Monitoring in private universities in Western Uganda

**Qualitative Findings**

Deans indicated that academic staff have mixed reactions to performance monitoring:

Some staff see it as a useful tool for improvement.

Others perceive it as a form of micromanagement or surveillance, especially if feedback is not constructive or is poorly communicated.

“Some staff take monitoring positively when it’s transparent, but others fear it’s a way to punish them.”

Objective v: To examine the relationship between Performance Monitoring and Academic Staff Performance in private universities in Western Uganda

**Qualitative Findings:**

Deans emphasized that performance monitoring helps staff stay focused and motivated. Regular feedback and evaluations improve teaching quality and research output.

“Performance monitoring keeps staff accountable and gives them direction for improvement.”

## Data Availability

**Underlying data is available -OSF repository** DOI: https://doi.org/10.17605/OSF.IO/TR8S7 ( Silaji *et al.*, 2025a) CC-By Attribution 4.0 International
