# Supplementary material for: Organizational Structure, Performance Monitoring, and Academic Staff Performance in Selected Private Chartered Universities: A Qualitative Study
Source: Open Res Afr. 2026 Jan 27;8:7. Originally published 2025 Jul 25. [Version 4] doi: 10.12688/openresafrica.15891.4 (PMC12854204; doi:10.12688/openresafrica.15891.4)
Supplement: Peer review history

# Reviewer report: Reviewer response for version 3

**DOI:** 10.21956/openresafrica.17516.r33424

**Published:** 2026-01-07

**Authors:** Hisham Al Ghunaimi

**Affiliations:** 1 A’Sharqiyah University, Ibra, Oman

This study investigates how organisational structure and performance monitoring influence academic staff performance in selected private chartered universities in Western Uganda. Using a qualitative phenomenological design, the authors draw on in-depth interviews with academic leaders to explore governance arrangements, quality assurance practices, monitoring systems, staff perceptions, and institutional support mechanisms. The revised version of the manuscript incorporates a strengthened theoretical framework grounded in Contingency Theory, Herzberg’s Motivation–Hygiene Theory, and Organizational Justice Theory. This theoretical integration allows the findings to be interpreted beyond description, offering explanatory insight into how structural rigidity, compliance-driven monitoring, and perceived fairness shape motivation, morale, and performance outcomes within private higher education institutions.

 Overall, the article provides a timely and policy-relevant contribution to African higher education governance literature and demonstrates sound qualitative methodology and analytical maturity.

**Evaluation in Relation to the Review Questions**

**1. Clarity, Accuracy, and Use of Literature – Yes**

 The manuscript is clearly written, logically structured, and appropriately grounded in both classical and contemporary literature on organizational structure, performance management, and higher education governance. The background, theoretical framing, and discussion sections demonstrate awareness of relevant African and international scholarship. The conceptual framing is coherent, and the literature is used not merely for citation purposes but as an interpretive lens for explaining the findings.

 No major corrections are required in this area.

**2. Appropriateness of Study Design and Technical Soundness – Yes**

 The phenomenological qualitative design is appropriate for the research objectives, which aim to explore lived experiences, perceptions, and institutional practices. The sampling strategy, data collection procedures, thematic analysis approach, and trustworthiness measures are adequately justified and clearly reported. Ethical approvals are explicitly documented, further strengthening the technical integrity of the study.

**3. Adequacy of Methodological Detail for Replication – Yes**

 The manuscript provides sufficient methodological detail to allow conceptual replication. The interview procedures, participant selection, analytical framework, and coding approach are transparently described. A researcher wishing to conduct a similar qualitative inquiry in another context would be able to follow the same methodological logic.

**4. Statistical Analysis – Not Applicable**

 The study is purely qualitative and does not involve statistical analysis. The absence of statistical testing is appropriate given the phenomenological research design.

**5. Availability of Source Data – Partly**

 While the manuscript includes extensive verbatim quotations and rich thematic reporting, it does not indicate whether anonymized interview transcripts, coding frameworks, or supplementary qualitative data are publicly available in a repository.

**This is the only area requiring formal improvement to strengthen the manuscript’s scientific transparency and reproducibility.**

**Required improvement:**

 The authors should include a brief data availability statement specifying one of the following:

- Whether anonymized interview transcripts and coding structures are available in an open repository, and where they can be accessed; or
- If public sharing is not possible due to ethical or institutional restrictions, a clear statement explaining the restrictions and conditions under which data may be accessed upon reasonable request.

This addition will ensure compliance with open research principles and enhance the study’s credibility.

**6. Support for Conclusions – Yes**

 The conclusions are well supported by the empirical findings and theoretical interpretation. The discussion logically synthesizes the qualitative evidence with the adopted theoretical lenses, clearly explaining how centralized governance, compliance-oriented monitoring, and perceived justice shape motivation and performance. The policy and institutional implications are reasonable and consistent with the data presented.

**Final Recommendation**

 The manuscript is scientifically sound, methodologically rigorous, and analytically well developed. It makes a meaningful contribution to higher education governance research in the African context.

**The article is suitable for approval, subject to one minor but important revision: the inclusion of a clear data availability statement addressing the accessibility or restrictions of the underlying qualitative data.**

 Once this minor transparency requirement is addressed, the article will fully meet open research and indexing standards.

Is the work clearly and accurately presented and does it cite the current literature?

Yes

If applicable, is the statistical analysis and its interpretation appropriate?

Not applicable

Are all the source data underlying the results available to ensure full reproducibility?

Partly

Is the study design appropriate and is the work technically sound?

Yes

Are the conclusions drawn adequately supported by the results?

Yes

Are sufficient details of methods and analysis provided to allow replication by others?

Yes

Reviewer Expertise:

Business management, Organizational Behavior & Organizational Justice, Motivation, and Work Engagement

I confirm that I have read this submission and believe that I have an appropriate level of expertise to confirm that it is of an acceptable scientific standard, however I have significant reservations, as outlined above.

## References

[1] : A reinterpretation of the direction of effects in studies of socialization. *Psychological Review* .1968;75(2) : 10.1037/h0025583 81-95 10.1037/h0025583 4870552

# Response

**Published:** 2026-01-07

**Authors:** TURYAMUREEBA SILAJI

**Our appreciation** Dear Reviewer, We sincerely thank you for your thorough, constructive, and insightful evaluation of our manuscript entitled *“Organisational Structure, Performance Monitoring, and Academic Staff Performance in Private Chartered Universities in Western Uganda.”* We are deeply appreciative of the time and scholarly care you devoted to reviewing our work. We are particularly grateful for your positive assessment of the study’s conceptual clarity, methodological rigor, and theoretical integration. Your recognition of the strengthened theoretical framework grounded in Contingency Theory, Herzberg’s Motivation–Hygiene Theory, and Organizational Justice Theory affirmed our intention to move beyond descriptive analysis toward a more explanatory and analytically mature interpretation of the findings. We also value your acknowledgement of the study’s relevance to African higher education governance and its potential policy contribution. We appreciate your confirmation that the qualitative phenomenological design, sampling strategy, data collection procedures, and analytical approach are appropriate, technically sound, and sufficiently detailed to allow conceptual replication. Your affirmation of the study’s ethical integrity and trustworthiness further strengthens our confidence in the robustness of the research. Regarding the single area identified for improvement **availability of source data** we fully agree with your recommendation. In response, we have added a clear **Data Availability Statement** to the revised manuscript. Silaji, T., Bagiwa, z. l., & Muhammad, T. (2026, January 7). Organizational Structure, Performance Monitoring, and Academic Staff Performance in Selected Private Chartered Universities: A Qualitative Study. https://doi.org/10.17605/OSF.IO/TR8S7 This statement specifies whether anonymized interview transcripts and coding frameworks can be accessed through an appropriate repository or, where ethical and institutional constraints apply, explains the conditions under which the data may be made available upon reasonable request. We believe this addition enhances transparency, aligns the manuscript with open research principles, and strengthens its credibility. Once again, we sincerely thank you for your constructive feedback, encouragement, and professional guidance. Your review has contributed meaningfully to improving the quality, clarity, and transparency of the manuscript. Yours faithfully. Corresponding author

**Turyamureeba Silaji**

 On behalf of the authors

# Reviewer report: Reviewer response for version 2

**DOI:** 10.21956/openresafrica.17451.r32808

**Published:** 2025-11-21

**Authors:** Muhammad Zaurez Afshar

**Affiliations:** 1 Lincoln University College, Petaling Jaya, Selangor, Malaysia

The paper is well-titled, concise, and reflective of its content. It effectively conveys the scope of inquiry and identifies the central variables—organizational structure, performance monitoring, and academic staff performance—within the context of private universities in Uganda. The revised version (v2) shows significant improvement in structure, thematic flow, and analytical clarity.

**Theoretical Framework**

 The manuscript references key organizational theorists (e.g., Mintzberg, Armstrong) but would benefit from deeper theoretical grounding. Integrating models such as:

- **Contingency Theory** – to explain how organizational design fits institutional contexts.
- **Herzberg’s Motivation-Hygiene Theory** – to interpret intrinsic and extrinsic motivators.
- **Organizational Justice Theory** – to examine fairness and perception of monitoring systems.

This would elevate the analytical rigor and situate findings within an established theoretical discourse.

**Data Analysis and Findings**

 The thematic analysis is comprehensive and logically sequenced through five major themes:

1. Organizational Structure
2. Performance Monitoring
3. Relationship and Perception of Staff
4. Academic Staff Performance
5. General Feedback and Recommendations

The presentation of verbatim excerpts enhances credibility and gives voice to participants. The discussion effectively connects findings to existing literature, showing both convergence and divergence with prior studies.

**Suggestion:** Some sections are descriptive; integrating interpretive commentary (i.e., *why* and *how* certain structures impact morale or productivity) would deepen the analytical insight.

**Presentation and Structure**

 The paper’s organization is coherent and professional. The use of subheadings (themes and subthemes) makes the narrative easy to follow. However:

- Figures (1–5) are conceptually useful but could be enhanced graphically to depict relationships among organizational structure, monitoring systems, and performance outcomes.

**Summary**

 This study is a well-conceived and ethically conducted qualitative inquiry that significantly contributes to understanding governance, monitoring, and academic performance in private higher education institutions. It combines academic rigor with practical applicability and demonstrates strong potential for indexing in academic and policy-oriented forums.

**Recommended improvements** include stronger theoretical framing, enhanced analytical interpretation, and refined presentation. With these refinements, the paper will stand as a strong reference for higher education governance research in developing contexts.

Is the work clearly and accurately presented and does it cite the current literature?

Yes

If applicable, is the statistical analysis and its interpretation appropriate?

Yes

Are all the source data underlying the results available to ensure full reproducibility?

Yes

Is the study design appropriate and is the work technically sound?

Yes

Are the conclusions drawn adequately supported by the results?

Yes

Are sufficient details of methods and analysis provided to allow replication by others?

Yes

Reviewer Expertise:

Organizational Resilience, Management, Finance

I confirm that I have read this submission and believe that I have an appropriate level of expertise to confirm that it is of an acceptable scientific standard.

# Response

**Published:** 2025-11-22

**Authors:** TURYAMUREEBA SILAJI

Dear Reviewer, Thank you for your thorough and insightful review of our manuscript, "Organizational Structure, Performance Monitoring, and Academic Staff Performance in Selected Private Chartered Universities: A Qualitative Study." We appreciate your validation of the paper's title, methodology, and ethical conduct. Your suggestions for enhancing the theoretical framework and deepening the analytical interpretation were critical and have led to substantial improvements, moving the paper from a descriptive presentation of findings to a robust theoretical discourse. Below, we detail how we addressed each of your specific points. 1. Theoretical Framework (Integration of Key Models)

**Reviewer Comment:** *The manuscript references key organizational theorists (e.g., Mintzberg, Armstrong) but would benefit from deeper theoretical grounding. Integrating models such as: Contingency Theory, Herzberg’s Motivation-Hygiene Theory, Organizational Justice Theory. This would elevate the analytical rigor and situate findings within an established theoretical discourse.*

**Our Response:** We fully agree and have significantly strengthened the theoretical grounding. We introduced a new, dedicated subsection ( **Section 1.2 Theoretical Grounding**) in the Introduction to frame the study using the suggested models. Theory Integrated Application in Manuscript **Contingency Theory** Used to interpret **Organizational Structure**. It explains why the centralized structure persists—as a necessary **contingency response** to regulatory and financial pressures—while simultaneously creating **misalignment** with the operational need for academic autonomy. **Herzberg’s Motivation-Hygiene Theory** Used to interpret **Performance Monitoring**. We argue that the compliance-based system and timely salary payments function as **Hygiene Factors** (preventing dissatisfaction), but fail to provide **Motivator Factors** (achievement, recognition), explaining the reported lack of "inner drive." **Organizational Justice Theory** Used to interpret **Staff Perception**. This model breaks down resentment into issues of **Procedural Justice** (unclear criteria, biased student feedback) and **Distributive Justice** (punitive salary deductions), explaining how perceived unfairness mediates the link between monitoring and performance.

 2. Data Analysis and Findings (Integrating Interpretive Commentary)

**Reviewer Comment:** *Suggestion: Some sections are descriptive; integrating interpretive commentary (i.e., why and how certain structures impact morale or productivity) would deepen the analytical insight.*

**Our Response:** We revised the analytical summary paragraphs within the Findings and Discussion sections (specifically the analysis for Themes 1, 2, and 3) to move beyond description and provide the requested "why" and "how."

- **Organizational Structure (Theme 1):** The analysis now explicitly states **how** the structure inhibits efficiency by creating a "misalignment" between centralized control and academic autonomy, interpreting this through **Contingency Theory**.
- **Performance Monitoring (Themes 2 & 3):** The analysis now explains **why** compliance monitoring creates resentment. This is attributed to the lack of **Motivator Factors** (Herzberg) and the violation of **Procedural** and **Distributive Justice** (Organizational Justice Theory), which directly lowers morale and commitment.

3. Presentation and Structure (Figure Enhancement)

**Reviewer Comment:** *Figures (1–5) are conceptually useful but could be enhanced graphically to depict relationships among organizational structure, monitoring systems, and performance outcomes.*

**Our Response:** We have addressed this by consolidating the conceptual representation into a single, comprehensive diagram.

- We created a new figure, **Figure 1: Conceptual Framework for Academic Staff Performance,** which has been placed after the Theoretical Grounding section.
- This figure clearly depicts the relationships between **Organizational Structure**, **Performance Monitoring**, and **Academic Staff Performance**, and visually embeds the three integrated theories ( **Contingency, Herzberg, and Organizational Justice**) as the critical mediators and drivers of these relationships. This provides a clear, high-level framework for the entire paper's analysis.

We believe that these refinements—particularly the strengthened theoretical framing and the resulting deeper analytical interpretation—have significantly elevated the academic rigor and practical applicability of the manuscript. We are confident that the paper now stands as a robust reference for higher education governance research.

 Sincerely,

 Turyamureeba Silaji

 Corresponding Author

 Kampala International University

# Reviewer report: Reviewer response for version 1

**DOI:** 10.21956/openresafrica.17066.r32402

**Published:** 2025-10-10

**Authors:** Rajindra S.E

**Affiliations:** 1 Universitas Muhammadiyah Palu, Palu, Indonesia

The Findings section (Section 4) should be improved.

 Authors should use a flowing, cohesive thematic narrative rather than using the Q&A session for interview questions. Use descriptive thematic subheadings (e.g., "Implications of Centralization for Operational Efficiency") rather than using interview questions as subheadings.

 Simplify Numbering: Simplify complex numbering systems (e.g., 4.2.4.1) into a more standard structure (4.1, 4.1.1, 4.2, etc.) to improve readability. Ensure research findings are presented in the following order (Structure, Monitoring, Performance, Recommendations).

 Substantial Improvements.

 Authors should add a new subsection within the research findings that explicitly addresses and supports the Abstract's claim regarding the relationship between dean qualifications and inclusive leadership styles and stronger faculty performance. If there is insufficient interview data to support these findings, these claims should be removed from the Abstract and Conclusion.

 Strengthen the discussion regarding the conflict between the overemphasis on attendance-based compliance (90%) and its negative impact on intrinsic motivation and work-life balance. The analysis should thoroughly examine how a robust system and the use of sanctions (salary reductions) can improve the culture.

 A conclusion should be added to this article to provide a comprehensive overview. We found no explicit conclusion in the article, and the lack of qualitative data on the performance monitoring theme limited our review. However, there is an overview of the findings in the abstract.

Is the work clearly and accurately presented and does it cite the current literature?

Yes

If applicable, is the statistical analysis and its interpretation appropriate?

I cannot comment. A qualified statistician is required.

Are all the source data underlying the results available to ensure full reproducibility?

Partly

Is the study design appropriate and is the work technically sound?

Partly

Are the conclusions drawn adequately supported by the results?

Partly

Are sufficient details of methods and analysis provided to allow replication by others?

Yes

Reviewer Expertise:

Managemen

I confirm that I have read this submission and believe that I have an appropriate level of expertise to confirm that it is of an acceptable scientific standard, however I have significant reservations, as outlined above.

# Response

**Published:** 2025-10-11

**Authors:** TURYAMUREEBA SILAJI

**Response to Reviewer Comments** Dear Reviewer, We sincerely thank you for your thoughtful and constructive feedback on our manuscript. We greatly appreciate your detailed suggestions, which have been invaluable in guiding substantial improvements to our work. Below, we provide a point-by-point response to your comments.

**1. Improving the Findings Section (Section 4)**

 We have revised Section 4 to ensure a flowing and cohesive thematic narrative rather than presenting the findings in a Q&A format. The revised section now uses descriptive thematic subheadings that clearly reflect the content, such as *“Implications of Centralization for Operational Efficiency”* and *“Performance Monitoring Practices and Faculty Motivation”*. This approach enhances readability and allows the reader to follow the logical progression of the findings.

**2. Simplifying Numbering**

 We have simplified the complex numbering system (previously 4.2.4.1) to a standard hierarchical structure (e.g., 4.1, 4.1.1, 4.2) to improve clarity and ease of navigation through the text.

**3. Presentation Order of Findings**

 The research findings are now presented in the order suggested:

- **Organizational Structure**
- **Performance Monitoring**
- **Academic Staff Performance**
- **Conclusion **
- **Recommendations**

This structured approach ensures a logical flow and aligns the discussion with the objectives of the study.

**4. Dean Qualifications, Inclusive Leadership, and Faculty Performance**

 We have added a new subsection explicitly addressing the relationship between dean qualifications, inclusive leadership styles, and faculty performance. Where sufficient qualitative evidence exists, we have provided thematic analysis to support the Abstract’s claim. In instances where interview data were insufficient, the Abstract and Conclusion have been revised to accurately reflect the findings, avoiding unsupported claims.

**5. Attendance-Based Compliance and Its Implications**

 We have strengthened the discussion regarding the conflict between the overemphasis on attendance-based compliance (90%) and its negative effects on intrinsic motivation and work-life balance. The revised analysis explores how a robust performance monitoring system, coupled with appropriate sanctions (e.g., salary reductions), can cultivate a culture of accountability while supporting staff engagement and well-being. **6. Conclusion**

 A comprehensive Conclusion section has now been added to provide a synthesis of the study’s main findings, implications, and recommendations. This conclusion integrates both quantitative and qualitative insights, ensuring alignment with the study’s objectives and the evidence presented. We are confident that these revisions address your concerns and enhance the clarity, cohesion, and rigor of our manuscript. We greatly appreciate your time and constructive feedback, which have significantly strengthened the quality of our work. Sincerely,

   Dr. Silaji Turyamureeba (Corresponding Author) On behalf of all Co-Authors

# Response

**Published:** 2025-10-14

**Authors:** TURYAMUREEBA SILAJI

**RESPONSE TO PEER REVIEWER COMMENTS**

 Dear reviewer,

 Thank you so much, kindly receive our responses bellow;

**Reviewer Comment: Improvement of the Findings Section** The reviewer recommends that the Findings section (Section 4) be improved by replacing the Q&A presentation style with a cohesive thematic narrative. They also suggest using descriptive thematic subheadings (e.g., *Implications of Centralization for Operational Efficiency*) instead of interview questions as subheadings.

**Author Response:** We appreciate this valuable suggestion. The Findings section has been thoroughly revised to present a flowing, cohesive thematic narrative rather than a question-and-answer format. Each theme is now discussed under clear, descriptive subheadings that reflect the content and significance of the findings. For example, new thematic subheadings such as *“Implications of Centralization for Operational Efficiency,”* *“Effects of Hierarchical Monitoring on Academic Staff Engagement,”* and *“Balancing Compliance and Autonomy in Performance Monitoring”* have been introduced. This restructuring enhances readability and analytical coherence, aligning the qualitative presentation with best practices in thematic reporting.

**Reviewer Comment: Simplify Numbering System and Order of Findings** The reviewer recommends simplifying the complex numbering system (e.g., 4.2.4.1) to a more standard and readable format (4.1, 4.1.1, 4.2, etc.). The reviewer also suggests ensuring the findings are presented in the logical order: *Structure → Monitoring → Performance → Recommendations.* **Author Response:** We have simplified the numbering system throughout Section 4 for clarity and consistency. The findings are now organized according to the suggested sequence, beginning with organizational structure, followed by performance monitoring, academic staff performance, and concluding with recommendations. This new structure improves the logical flow and enhances the reader’s understanding of the progression from institutional structures to staff performance outcomes.

**Reviewer Comment: Add Subsection on Dean Qualifications and Inclusive Leadership** The reviewer recommends adding a new subsection within the research findings to explicitly address the relationship between deans’ qualifications, inclusive leadership styles, and faculty performance, as referenced in the Abstract. If there is insufficient data to support this, the claims should be removed from the Abstract and Conclusion.

**Author Response:** We thank the reviewer for highlighting this important point. Upon review of the qualitative and quantitative data, sufficient evidence was found to support a subsection on the relationship between deans’ academic qualifications, inclusive leadership practices, and faculty performance outcomes. This new subsection has been added under the Findings section to substantiate the claim made in the Abstract. The analysis demonstrates that deans with higher academic qualifications and participatory leadership styles tend to foster stronger faculty motivation and performance. Where evidence was limited, the statements in the Abstract and Conclusion were refined to maintain accuracy and avoid overgeneralization.

**Reviewer Comment: Strengthen Discussion on Compliance and Motivation** The reviewer advises that the discussion should more deeply examine the conflict between overemphasis on attendance-based compliance (90%) and its negative effects on intrinsic motivation and work-life balance. The reviewer also recommends analyzing how a robust system, including sanctions (e.g., salary reductions), can shape institutional culture.

**Author Response:** We agree with the reviewer’s insightful comment and have expanded the discussion accordingly. The revised discussion now critically explores the tension between strict attendance-based monitoring and intrinsic motivation among academic staff. We have analyzed how overreliance on compliance indicators may undermine creativity and autonomy, drawing on relevant recent literature (2023–2025). The expanded section also discusses how transparent and fair enforcement mechanisms—such as clearly communicated sanctions—can help reinforce accountability while maintaining staff morale and a positive institutional culture.

**Reviewer Comment: Add a Conclusion Section** The reviewer notes the absence of an explicit conclusion and recommends adding one to provide a comprehensive overview of the findings. They also observed limited qualitative data on the performance monitoring theme. **Author Response:** We appreciate this observation and have now included a dedicated Conclusion section at the end of the manuscript. The new conclusion synthesizes the key findings from both quantitative and qualitative analyses, highlights the study’s theoretical and practical implications, and suggests directions for future research. While the qualitative data on performance monitoring were limited, we have reanalyzed and integrated relevant interview excerpts to strengthen the interpretation of this theme. The conclusion now aligns closely with the study’s objectives and provides a coherent summary for readers.

**Summary of Revisions** In response to the reviewer’s comments, the manuscript has undergone substantial improvement. The Findings section has been rewritten in a thematic and cohesive style with simplified numbering and clearer structure. A new subsection on deans’ qualifications and inclusive leadership has been added, and the discussion has been expanded to address motivational conflicts arising from compliance-based monitoring.

 Finally, a comprehensive conclusion has been added to summarize findings, implications, and future directions. We believe these revisions significantly enhance the clarity, rigor, and coherence of the paper.

 Thanks Sincerely,

 Turyamureeba Silaji silaji.turyamureeba@studwc.kiu.ac.ug

Corresponding Author Kampala Internal University
