# Supplementary material for: Organizational Structure, Performance Monitoring, and Academic Staff Performance in Selected Private Chartered Universities: A Qualitative Study
Source: Open Res Afr. 2025 Oct 17;8:7. Originally published 2025 Jul 25. [Version 2] doi: 10.12688/openresafrica.15891.2 (PMC12854204; doi:10.12688/openresafrica.15891.2)
Supplement: Peer review history

# Response

**Published:** 2025-10-14

**Authors:** TURYAMUREEBA SILAJI

**RESPONSE TO PEER REVIEWER COMMENTS**

 Dear reviewer,

 Thank you so much, kindly receive our responses bellow;

**Reviewer Comment: Improvement of the Findings Section** The reviewer recommends that the Findings section (Section 4) be improved by replacing the Q&A presentation style with a cohesive thematic narrative. They also suggest using descriptive thematic subheadings (e.g., *Implications of Centralization for Operational Efficiency*) instead of interview questions as subheadings.

**Author Response:** We appreciate this valuable suggestion. The Findings section has been thoroughly revised to present a flowing, cohesive thematic narrative rather than a question-and-answer format. Each theme is now discussed under clear, descriptive subheadings that reflect the content and significance of the findings. For example, new thematic subheadings such as *“Implications of Centralization for Operational Efficiency,”* *“Effects of Hierarchical Monitoring on Academic Staff Engagement,”* and *“Balancing Compliance and Autonomy in Performance Monitoring”* have been introduced. This restructuring enhances readability and analytical coherence, aligning the qualitative presentation with best practices in thematic reporting.

**Reviewer Comment: Simplify Numbering System and Order of Findings** The reviewer recommends simplifying the complex numbering system (e.g., 4.2.4.1) to a more standard and readable format (4.1, 4.1.1, 4.2, etc.). The reviewer also suggests ensuring the findings are presented in the logical order: *Structure → Monitoring → Performance → Recommendations.* **Author Response:** We have simplified the numbering system throughout Section 4 for clarity and consistency. The findings are now organized according to the suggested sequence, beginning with organizational structure, followed by performance monitoring, academic staff performance, and concluding with recommendations. This new structure improves the logical flow and enhances the reader’s understanding of the progression from institutional structures to staff performance outcomes.

**Reviewer Comment: Add Subsection on Dean Qualifications and Inclusive Leadership** The reviewer recommends adding a new subsection within the research findings to explicitly address the relationship between deans’ qualifications, inclusive leadership styles, and faculty performance, as referenced in the Abstract. If there is insufficient data to support this, the claims should be removed from the Abstract and Conclusion.

**Author Response:** We thank the reviewer for highlighting this important point. Upon review of the qualitative and quantitative data, sufficient evidence was found to support a subsection on the relationship between deans’ academic qualifications, inclusive leadership practices, and faculty performance outcomes. This new subsection has been added under the Findings section to substantiate the claim made in the Abstract. The analysis demonstrates that deans with higher academic qualifications and participatory leadership styles tend to foster stronger faculty motivation and performance. Where evidence was limited, the statements in the Abstract and Conclusion were refined to maintain accuracy and avoid overgeneralization.

**Reviewer Comment: Strengthen Discussion on Compliance and Motivation** The reviewer advises that the discussion should more deeply examine the conflict between overemphasis on attendance-based compliance (90%) and its negative effects on intrinsic motivation and work-life balance. The reviewer also recommends analyzing how a robust system, including sanctions (e.g., salary reductions), can shape institutional culture.

**Author Response:** We agree with the reviewer’s insightful comment and have expanded the discussion accordingly. The revised discussion now critically explores the tension between strict attendance-based monitoring and intrinsic motivation among academic staff. We have analyzed how overreliance on compliance indicators may undermine creativity and autonomy, drawing on relevant recent literature (2023–2025). The expanded section also discusses how transparent and fair enforcement mechanisms—such as clearly communicated sanctions—can help reinforce accountability while maintaining staff morale and a positive institutional culture.

**Reviewer Comment: Add a Conclusion Section** The reviewer notes the absence of an explicit conclusion and recommends adding one to provide a comprehensive overview of the findings. They also observed limited qualitative data on the performance monitoring theme. **Author Response:** We appreciate this observation and have now included a dedicated Conclusion section at the end of the manuscript. The new conclusion synthesizes the key findings from both quantitative and qualitative analyses, highlights the study’s theoretical and practical implications, and suggests directions for future research. While the qualitative data on performance monitoring were limited, we have reanalyzed and integrated relevant interview excerpts to strengthen the interpretation of this theme. The conclusion now aligns closely with the study’s objectives and provides a coherent summary for readers.  

**Summary of Revisions** In response to the reviewer’s comments, the manuscript has undergone substantial improvement. The Findings section has been rewritten in a thematic and cohesive style with simplified numbering and clearer structure. A new subsection on deans’ qualifications and inclusive leadership has been added, and the discussion has been expanded to address motivational conflicts arising from compliance-based monitoring.

 Finally, a comprehensive conclusion has been added to summarize findings, implications, and future directions. We believe these revisions significantly enhance the clarity, rigor, and coherence of the paper.

 Thanks Sincerely,

 Turyamureeba Silaji:silaji.turyamureeba@studwc.kiu.ac.ug

Corresponding Author Kampala Internal University
